# Treatment choice for first permanent molars affected with molar-incisor hypomineralization, in patients 7–8 years of age: a questionnaire study among Swedish general dentists, orthodontists, and pediatric dentists

**DOI:** 10.1007/s40368-023-00860-9

**Published:** 2024-02-05

**Authors:** A. Hajdarević, E. Čirgić, A. Robertson, N. Sabel, B. Jälevik

**Affiliations:** 1 Department of Pediatric Dentistry, Institute of Odontology, Sahlgrenska Academy, University of Gothenburg, Gothenburg, Sweden; 2 Clinic of Pediatric Dentistry, Public Dental Service, Region Västra Götaland, Gothenburg, Sweden; 3 Institute of Odontology, Sahlgrenska Academy, University of Gothenburg, Gothenburg, Sweden; 4 Folktandvården Björkekärr, Public Dental Service, Region Västra Götaland, Gothenburg, Sweden

**Keywords:** Dental enamel defect, Therapy option, General dentistry, Pediatric dentistry, Orthodontics, Index

## Abstract

**Purpose:**

The aim of this study was to investigate attitudes and preferred therapy choice for first permanent molars (FPM) with Molar-Incisor Hypomineralization (MIH).

**Methods:**

An online questionnaire was sent out to general dentists (*n* = 559) working in the Public Dental Service in Region Västra Götaland, orthodontists (*n* = 293), and pediatric dentists (*n* = 156) (members from each interest association), in Sweden. The questionnaire contained three parts: general questions regarding the respondents, patient cases, and general questions regarding extraction of FPMs with MIH. Statistics were carried out using Chi-squared tests, with a significance level of 5%.

**Results:**

A response rate of 36% was obtained. Orthodontists and pediatric dentists were more prone to extract FPMs with both moderate and severe MIH, compared to general dentists. When restoring FPMs with moderate MIH, resin composite was preferred. Compared to the general dentists, the pediatric dentists were more prone to choose glass-ionomer cement in the FPMs with severe MIH. The most common treatment choice for FPMs with mild MIH was fluoride varnish. “When root furcation is under development of the second permanent molar on radiographs” was chosen as the optimal time for extracting FPMs with severe MIH, and the general dentists based their treatment decisions on recommendations from a pediatric dentist.

**Conclusion:**

Extraction of FPMs with moderate and severe MIH is considered a therapy of choice among general dentists and specialists, and the preferred time of extraction is before the eruption of the second permanent molar.

**Supplementary Information:**

The online version contains supplementary material available at 10.1007/s40368-023-00860-9.

## Introduction

Recently erupted first permanent molars (FPM) are a burden for many children with pain caused by hypomineralized enamel, often with subsequent post-eruptive breakdown. This condition is named Molar-Incisor Hypomineralization (MIH) and may affect up to all four FPMs in varying degrees of severity, and in some cases, also involving the incisors (Weerheijm et al. 2001). Approximately 14% of children worldwide have FPMs with areas of hypomineralized enamel (Zhao et al. 2018; Schwendicke et al. 2018). Clinically, the affected teeth have white, white/yellow or brownish, well-defined opacities. In more severe cases, post-eruptive breakdown occurs due to porous enamel (Jälevik and Norén 2000). Despite extensive research, the etiology of MIH is still unclear (Garot et al. 2021; Lygidakis et al. 2022).

The condition causes problems for the child due to hypersensitivity, especially when consuming cold drinks and food, inhaling air, and tooth brushing (Raposo et al. 2019). In cases with post-eruptive breakdown, the risk of rampant caries increases (Americano et al. 2017; Villanueva Gutiérrez et al. 2019). Consequently, there may be a need for treatment shortly after tooth eruption, e.g., restorative or extraction. Both treatment choices find support in the literature (Bandeira Lopes et al. 2021; Elhennawy and Schwendicke 2016; Lygidakis et al. 2022).

A study by Jälevik and Klingberg (2002) showed that 9-year-old children with severe MIH received almost ten times more treatment for FPMs, compared to children without MIH. Children were exposed to repeated treatments of FPMs between the ages of 9–18 years, restorative as well as extractions, compared to patients without MIH (Jälevik and Klingberg 2012). Morphological changes in the enamel prisms cause difficultes in the etching and bonding of composite materials (Krämer et al. 2018; Jälevik et al. 2005). The deficient bonding and porous nature of enamel affected by MIH and restoration leads to an increased failure rate (Elhennawy and Schwendicke 2016).

Dental care in Sweden is free of charge until 19 years of age. The Public Dental Service is the main oral healthcare provider for children in Sweden, including care by general dentists and specialists. The Public Dental Service is run by 24 independent regions in Sweden, with Region Västra Götaland providing care for 96% of children and adolescents. Both orthodontic and pediatric dentists are registered specialties with 3 years compulsory, postgraduate education. Specialist consultations may be a part of the management of FPMs with MIH.

Until now, no study has evaluated attitudes, therapy choices, and the timing for extractions of FPMs with MIH, in Sweden. The aim of this study was to investigate the preferred therapy choice for first permanent molars with Molar-Incisor Hypomineralization (MIH), based on severity, among general dentists, orthodontists, and pediatric dentists. In addition, when choosing extraction therapy, the respondents were asked regarding the optimal time for possible extractions and what the respondents base their treatment decision on for first permanent molars with severe MIH.

## Materials and methods

### Participants

General dentists at the Public Dental Services, Region Västra Götaland, Sweden, (*n* = 559), as well as orthodontist members from the Swedish Association of Orthodontists (*n* = 293) and pediatric dentist members from the Swedish Society of Pediatric Dentistry (*n* = 156), were invited to participate in the study. An online questionnaire was sent out by e-mail in May 2021, with information regarding voluntary participation and questionnaire response anonymity. Two reminders were sent out after 4 weeks and 15 weeks. The questionnaire was initially substantiated using Google Forms, a web tool facilitating submission of answers from any browser. Due to the anonymity of the questionnaire, reminders were sent to all participants.

### Questionnaire

The questionnaire consisted of three parts.

The first part involved questions concerning the respondent’s professional background.

**Fig. 1:**
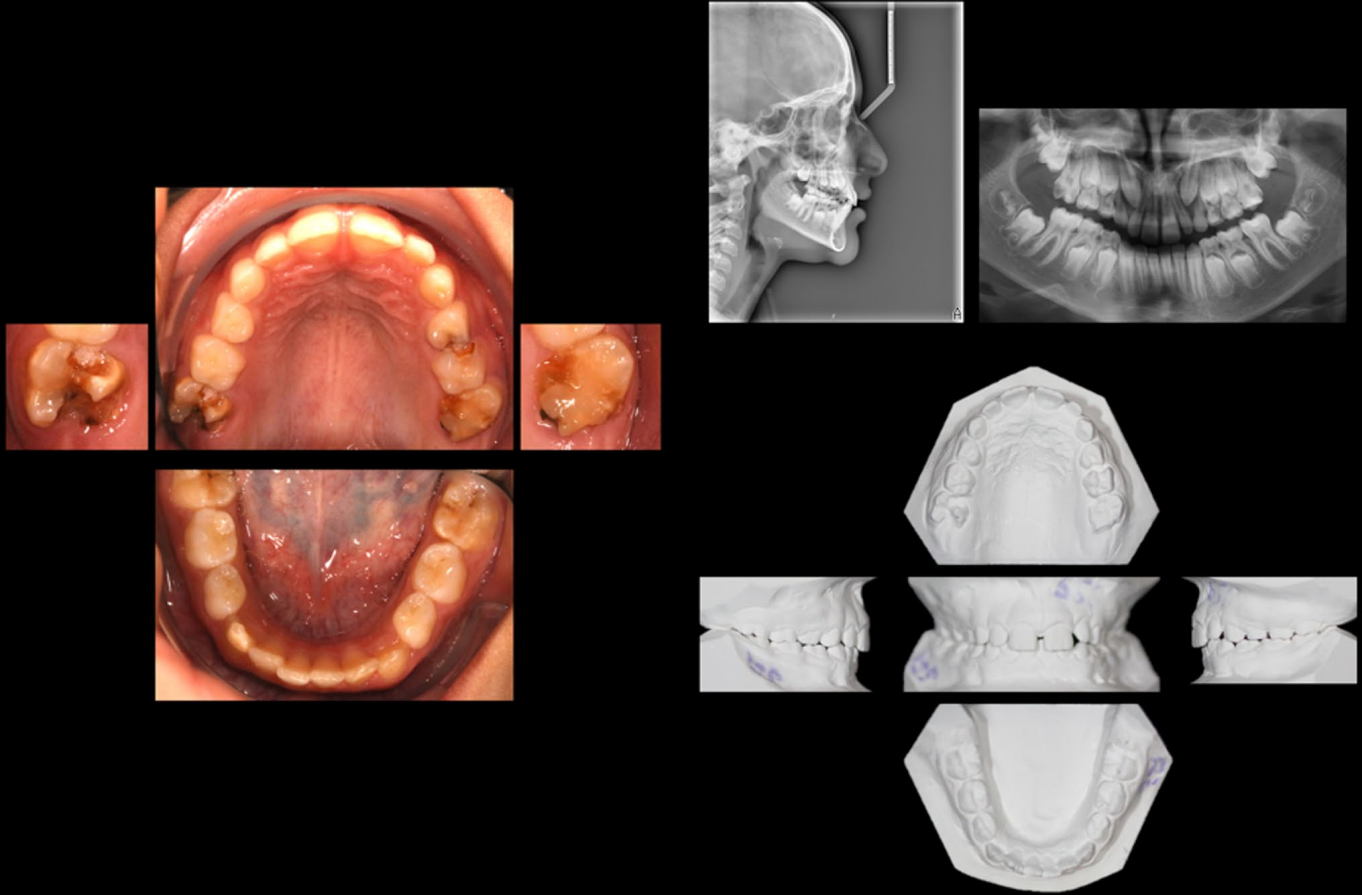
Patient case example including clinical intraoral photos, profile and panoramic radiographs, and photos of plaster models

**Fig. 2:**
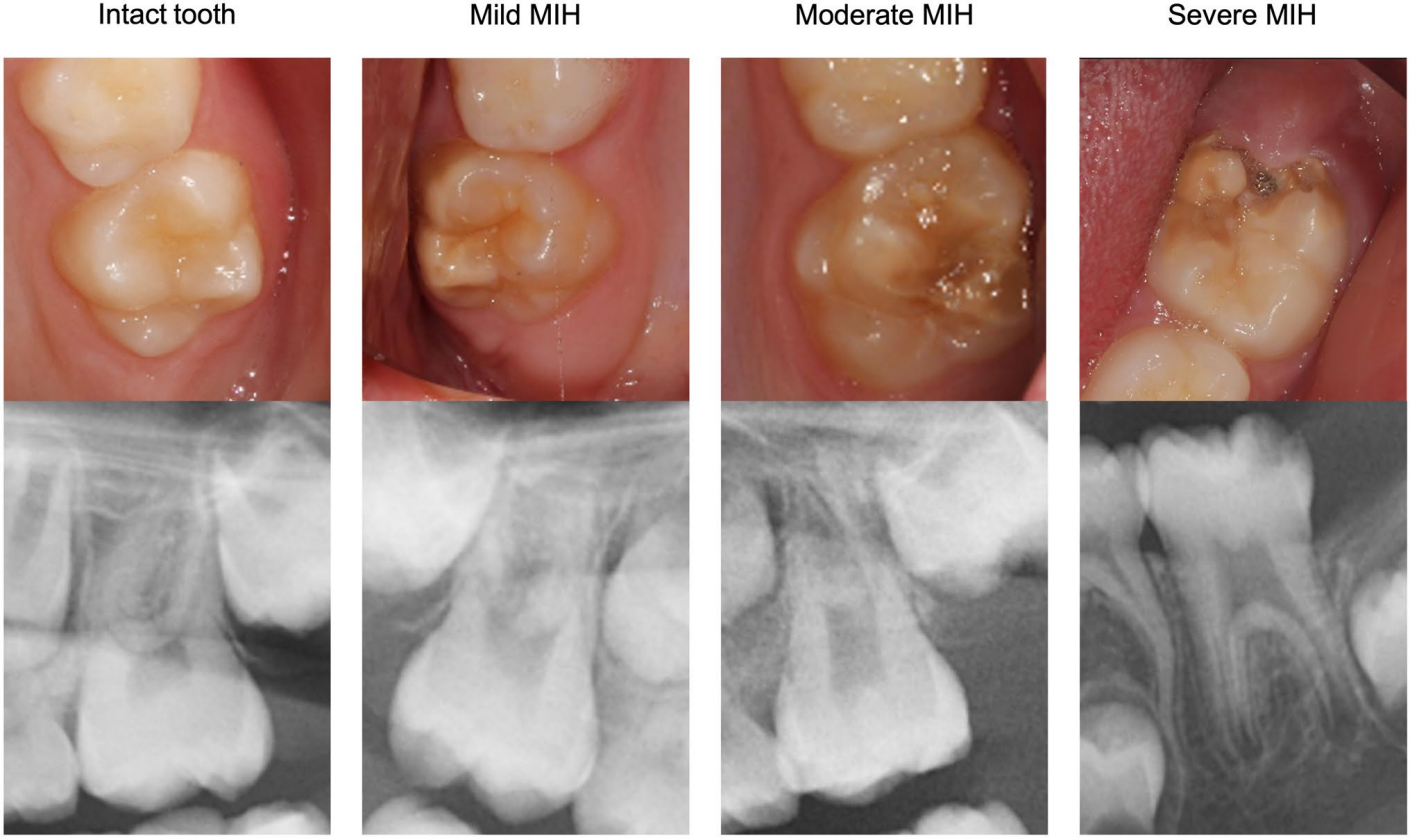
Simplified index of grading each first permanent molar, clinical and radiographic assessment. Intact tooth with sound enamel, mild MIH with demarcated opacities without enamel breakdown, moderate MIH with demarcated opacities with enamel breakdown or atypical restauration of ≤ 2 surfaces, and severe MIH with demarcated opacities with enamel breakdown or atypical restauration, involving > 2 surfaces and/or extensive lesion

In the second part, there were six cases of 7–8-year-old patients in the mixed dentition. The cases were presented by clinical intraoral photos (occlusal photos of the upper and lower arch), profile radiographs, panoramic radiographs, and photos of plaster models (Fig. 1). The cases presented various clinical manifestations of FPMs, with and without MIH, and varying orthodontic diagnoses. For each FPM, the dentists were asked to choose a treatment. Respondents were asked to assess the FPMs in each patient based on clinical photos, radiographs, and plaster models. No further information with respect to the patient was given. The FPMs were categorized into subgroups based on EAPD criteria (Jälevik 2010; Lygidakis et al. 2022) and expanded with an additional subgroup, moderate MIH (Table 1; Fig. 2).

**Table 1:** Assessment of each fully erupted first permanent molar, provided the patient/tooth has no other enamel defects, e.g., Amelogenesis Imperfecta, dental fluorosis, hypoplasia, or carious white spot lesions

MIH severity of the first permanent molar	Definition
Intact tooth	Sound enamel, or hypomineralization < 1 mm in diameter
Mild MIH	Demarcated opacities, without enamel breakdown
Moderate MIH	Hypomineralized enamel with enamel breakdown or atypical restauration, ≤ 2 surfaces
Severe MIH	Hypomineralized enamel with enamel breakdown or atypical restauration, > 2 surfaces and/or extensive lesion (> 2/3 of the depth of the dentin)

The gradings are summations of visual and radiographic assessments. The tooth is considered to have five surfaces: Occlusal, buccal, palatinal/lingual, mesial, and distal

The third part of the questionnaire consisted of two general questions regarding extraction treatment of FPMs with MIH. Swedish and English versions are included in Online Appendices 1–6.

The calibration between the authors—three pediatric dentists (N.S., A.R., B.J.), one orthodontist (E.C.), and one general dentist (A.H.)—was performed twice, by allowing the authors to grade each FPMs by clinical photos on a computer screen and radiographs, with an interval of 5 weeks. Of the 24 FPMs in the six cases, 6 teeth were classified as intact, 4 were diagnosed to be affected with mild MIH, 3 with moderate MIH, and 11 with severe MIH. An excellent degree of agreement was found between the authors’ measurements. The average measured Intraclass Correlation Coefficient (ICC) was 0.956, with a 95% confidence interval from 0.920 to 0.979, using Two-way Mixed, and absolute agreement.

### Ethical considerations

This study was approved by the Reference Group concerning the research project within the Public Dental Service, Region Västra Götaland, Sweden. The anonymized patient cases used in the questionnaire are derived from a larger, ongoing study (Hajdarević 2023), approved by the Swedish Ethical Review Authority in Gothenburg (Registration No: 352-15), where the patients and caregivers gave written consent.

### Statistical analysis

For some analyses, the treatment measures were divided into two subgroups, extraction and non-extraction treatment. Respondents categorized as specialists were the orthodontists and pediatric dentists. IBM SPSS Statistics version 28.0 (Statistical Package for the Social Sciences; SPSS, Chicago, IL, USA) was used for statistical analyses. Statistical evaluation was performed using Chi-squared tests. A significance level of 5% was used.

## Results

In total, 357 respondents filled out the questionnaire resulting in a response rate of 36%. Fifty-four percent of the respondents were general dentists, 28% were orthodontists, and 18% were pediatric dentists. Work experience was significantly shorter (*p* < 0.001) among the general dentists (mean ± SD, 13.4 ± 11.6 years), compared to the orthodontists (28.0 ± 11.2 years) and pediatric dentists (27.4 ± 11.3 years). No significant difference in work experience was seen between the orthodontists and pediatric dentists. The general dentists had less experience treating children and adolescents, compared to the orthodontists and pediatric dentists, showing a significant difference (*p* < 0.001; Table 2).

**Table 2:** General questions responded by general dentists, orthodontists, and pediatric dentists, respectively

	General dentists n (%)	Orthodontists n (%)	Pediatric dentists n (%)
Total respondents	192 (100)	101 (100)	64 (100)
Gender			
Female	142 (74)	53 (53)	48 (75)
Male	48 (25)	48 (47)	12 (19)
Did not state their gender	2 (1)	0 (0)	4 (6)
Years as a dentist			
< 5	54 (28)	2 (2)	0 (0)
5–10	50 (26)	11 (11)	1 (2)
> 10	88 (46)	88 (87)	63 (98)
Years as a specialist dentist			
< 5		15 (15)	14 (22)
5–10		16 (16)	19 (30)
> 10		70 (69)	31 (48)
Weekly work hours with child dental care			
< 10	45 (23)	8 (8)	8 (13)
10–19	94 (49)	13 (13)	3 (5)
20–30	37 (19)	37 (37)	20 (31)
> 30	16 (8)	36 (36)	26 (41)
Non-clinical duty	0 (0)	7 (7)	7 (11)

For the cases of FPMs with severe MIH, the general dentists preferred extraction in less than half of the cases, while most of the orthodontists and pediatric dentists selected extraction (*p* < 0.001; Table 3). Specialists (60%), who had worked for more than 10 years, preferred extraction significantly more often than the general dentists (47%) with the same period of experience (*p* < 0.001), while there was no difference between the groups who had worked 5–10 years. Among the general dentists, the number of years as a dentist did not influence the preference for extraction of FPMs with severe MIH. Of the respondents, all had at least once chosen extraction as therapy for FPMs with severe MIH. Choosing extraction of FPMs with severe MIH was significantly more common in the upper jaw (68%), compared to the lower jaw (32%; *p* < 0.001).

**Table 3:** Choice of extraction (Ex) for first permanent molar with MIH, answered by general dentists, orthodontists, and pediatric dentists, respectively

	General dentists		Orthodontists		Pediatric dentists		p value (Chi-square test)		
	Non-Ex n (%)	Ex n (%)	Non-Ex n (%)	Ex n (%)	Non-Ex n (%)	Ex n (%)	General dentists vs orthodontists	General dentists vs pediatric dentists	Orthodontists vs pediatric dentists
Moderate MIH	539 (93.6)	37 (6.4)	256 (84.5)	47 (15.5)	157 (81.8)	35 (18.2)	< 0.001	< 0.001	NS
Severe MIH	1106 (52.4)	1006 (47.6)	434 (39.1)	677 (60.9)	292 (41.5)	412 (58.5)	< 0.001	< 0.001	NS

Responses of Non-Ex versus Ex were analyzed with Chi-square test, between: general dentists and orthodontists, general dentists and pediatric dentists, and orthodontists and pediatric dentists. Non-Extraction (Non-Ex) treatment incorporates: restoration with glass-ionomers cement, restoration with composite resin, stainless steel crown, porcelain crown, inlay or onlay, fluoride varnish, and to await outcome

*NS* non-significant

When restoring FPMs with moderate enamel disintegration, the general dentists, as well as the pediatric dentists, preferred resin composite as a first-choice material (Table 4). In selecting whether to keep or extract the defective tooth, most of the respondents chose non-extraction therapy. However, when extraction was chosen, the orthodontists and pediatric dentists were significantly more likely to propose this therapy, compared to general dentists (Table 3). The specialists (16%), with a clinical experience of more than 10 years, were significantly more prone to extract moderate MIH, compared to general dentists (7%) with comparable experience. Of all respondents, less than 1% chose stainless-steel or porcelain crowns, inlay, or onlay as a treatment option for FPMs with moderate MIH.

**Table 4:** Preferred choice of material (resin composite or glass-ionomers cement (GIC)) when restoring first permanent molar with moderate and severe MIH, answered by general dentists and pediatric dentists, respectively

	General dentists		Pediatric dentists		p value (Chi-square test) General dentists vs pediatric dentists
	Composite resin n (%)	GIC n (%)	Composite resin n (%)	GIC n (%)	
Moderate MIH	199 (76.0)	63 (24.0)	79 (77.5)	23 (22.5)	NS

Responses were analyzed with Chi-square test regarding resin composite versus GIC, between general dentists and pediatric dentists

*NS* non-significant

When treating FPMs with mild MIH, fluoride varnish was the most common choice among the general and pediatric dentists, while the orthodontists preferred to await the outcome (*p* < 0.001; Table 5).

**Table 5:** Non-invasive treatment choice for first permanent molar with mild MIH

	General dentists		Orthodontists		Pediatric dentists		p value (Chi-square test)		
	Fluoride varnish n (%)	Await outcome n (%)	Fluoride varnish n (%)	Await outcome n (%)	Fluoride varnish n (%)	Await outcome n (%)	General dentists vs orthodontists	General dentists vs pediatric dentists	Orthodontists vs pediatric dentists
Mild MIH	460 (73.4)	167 (26.6)	124 (42.3)	169 (57.7)	113 (64.4)	62 (35.4)	< 0.001	NS	< 0.001

Non-invasive treatment incorporates fluoride varnish and to await the outcome, answered by general dentists, orthodontists, and pediatric dentists, respectively. Responses were analyzed with Chi-square test regarding fluoride varnish versus to await the outcome, between: General dentists and orthodontists, general dentists and pediatric dentists, and orthodontists and pediatric dentists

*NS* non-significant

Regarding the opinion of an optimal time for extraction of FPMs with severe MIH, approximately half of the respondents chose “when root furcation is under development of the second permanent molar on radiographs”. None of the respondents chose “when the second permanent molar has fully erupted”. Approximately two-fifths of the general dentists chose “pediatric dentist’s recommendation” for their treatment decision (Table 6).

**Table 6:** Primary preferred alternatives of the two general questions regarding extraction therapy for first permanent molars with MIH, responded by general dentists, orthodontists, and pediatric dentists, respectively

General questions and preferred alternatives	General dentists n (%)	Orthodontists n (%)	Pediatric dentists n (%)	p value (Chi-square test)		
				General dentists vs orthodontists	General dentists vs pediatric dentists	Orthodontists vs pediatric dentists
What is the optimal time for extraction of FPM with severe MIH?						
Alternatives						
Immediately upon diagnosis	19 (9.9)	11 (10.9)	17 (26.6)	NS	< 0.001	< 0.01
Between the ages of 8–9 years	35 (18.2)	9 (8.9)	8 (12.5)	< 0.05	NS	NS
Root furcation development of second permanent molar on radiographs	87 (45.3)	47 (46.5)	29 (45.3)	NS	NS	NS
When the second permanent molar has partly erupted	34 (17.7)	21 (20.8)	5 (7.8)	NS	NS	< 0.05
When the second permanent molar has fully erupted	0 (0)	0 (0)	0 (0)	N/A	N/A	N/A
Other	17 (8.9)	13 (12.9)	5 (7.8)			
What the respondents based their treatment decision on while choosing restorative or extraction therapy for FPMs with severe MIH						
Alternatives						
Clinical experience	42 (21.9)	56 (55.4)	30 (46.9)	< 0.001	< 0.001	NS
Research	8 (4.2)	8 (7.9)	26 (40.6)	NS	< 0.001	< 0.01
Local guidelines	8 (4.2)	2 (2.0)	0 (0.0)	N/A	N/A	NS
Recommendations from orthodontists	49 (25.5)	12 (11.9)	3 (4.7)	< 0.01	N/A	N/A
Recommendations from pediatric dentists	85 (44.3)	23 (22.8)	5 (7.8)	< 0.001	< 0.001	< 0.05

## Discussion

This study has shown that Swedish general dentists, orthodontists, and pediatric dentists chose extraction of permanent molars, with both moderate and severe MIH, as primary therapy. This is the first study examining attitudes of therapy choices for FPMs affected by MIH, and the timing for possible extraction among dental practitioners in Sweden. This knowledge is essential for the understanding of which measures need to be taken to improve oral health care and the management of FPMs with MIH.

Concerning the FPMs with moderate as well as severe MIH, the specialists were more prone to choose extraction therapy, compared to the general dentists. Specialists with long experience distinguished themselves by choosing extraction more often than the general dentists with a similar period of experience, in all probability due to the experience of failed restorations and poor, long-term prognosis. This is in congruence with a study with long-term perspective, where 50% of a group of 18-year-olds with MIH and restorative treatment of FPMs had defects and unacceptable restorations (Mejàre et al. 2005). Considering extraction before restorative treatment may be due to the experience of negative patient cooperation, seen as pain and difficulties with inadequate anesthesia, common problems during restorative treatments (Crombie et al. 2008) presumably due to subclinical pulp inflammation caused by enamel porosity (Rodd et al. 2007; Fagrell et al. 2008). Painful treatment and retreatment may in turn result in dental fear and behavior management problems in children with severe MIH (Jälevik and Klingberg 2002). Achieving a favourable result of space closure after extraction of FPMs with MIH is a suitable treatment plan and should be considered if the long-term prognosis of the restauration is uncertain (Lygidakis et al. 2022), which may be the respondent’s reason for choosing extraction in cases with severe MIH.

All respondents had chosen extraction at least once for severe MIH, which is in congruence with recently published Swedish National Guidelines, with a high degree of recommendation to extract FPMs with severe MIH for children between 6 and 11 years of age (Socialstyrelsen 2022a). Similarly, the Royal College of Surgeon of England Clinical Guidelines (RCSEng 2023) point out that extraction of FPMs should be considered in cases with questionable, long-term prognosis, and emphasizes that treatment-planning for the enforced extraction of FPMs can present a complex problem, particularly in the presence of an underlying malocclusion (Cobourne et al. 2014). A study exploring current attitudes regarding the management of compromised FPMs among British general dentists and specialists in pediatric dentistry, found a great variation between and within each professional group (Taylor et al. 2019). Although the RCSEng guidelines have been implemented in the United Arab Emirates, a survey of dentists showed that 85% still preferred conservative treatment over extraction of FPMs with severe MIH (Dastouri et al. 2020). Nevertheless, the recommendations of extracting FPMs with poor prognosis are also contradictory. E.g., the AAPD (American Academy of Pediatric Dentistry) guideline websites for “Pediatric restorative dentistry” (AAPD 2019) and “Pulp therapy for Primary and immature permanent teeth” (AAPD 2020), do not recommend extraction as a treatment alternative for permanent teeth with poor prognosis.

Among general dentists in the present study, an overwhelming majority based their treatment decision on recommendations from a specialist, primarily a pediatric dentist. This indicates that general dentists require support with decisions to extract in the permanent dentition at young ages. Two other studies also reported that general dentists prefer to send a referral to pediatric dentists for the management of FPMs with MIH (Skaare et al. 2021; Hussein et al. 2014). Another study showed that two-thirds of Norwegian general dentists had extracted FPMs due to MIH, and found that orthodontics had been consulted in nearly all of those cases (Kopperud et al. 2016). Studies dealing with dentists’ perception of MIH conclude that their knowledge concerning treatment is limited (Seremidi et al. 2022; Delgado et al. 2022; Hamza et al. 2021).

Almost half of the respondents thought that the optimal time for extraction of FPMs was when root furcation development of the second permanent molar could be seen on radiographs. Optimal timing of extraction may allow space closure by the mesial movement of the second permanent molar (Cobourne et al. 2014; Saber et al. 2018). However, a systematic review aiming to identify the ideal time for extraction of FPMs and reducing a future need for orthodontic treatment has concluded: despite that extraction of FPMs might be clinically indicated, there is yet minor scientific evidence about the ideal extraction timing to minimize unwanted negative effects, such as remaining space, tipping and/or rotation of the second permanent molar (Eichenberger et al. 2015; Hatami and Dreyer 2019). Teo et al. (2013) showed an 80–88% success rate for complete space closure after extraction of FPMs within a follow-up period of 5 years. The extraction of the FPM was performed while the second permanent molar was in the Demirjian developmental stages E and F, implying the early, respectively, the late bifurcation development of the root (Demirjian et al. 1973). A systematic review concluded that there is yet no scientific evidence to support an ideal time for extraction of FPMs with poor prognosis (Wu et al. 2017). However, the ideal time for extraction of affected FPMs has been reported to be between 8 and 10 years of age (Thilander and Skagius 1970), though this does not guarantee complete spontaneous space closure (Ashley and Noar 2019). There are studies (Jälevik and Möller 2007; Mejàre et al. 2005) which have shown good subjective results in patients after extraction of FPMs with MIH, over time. Another factor that is decisive for good space closure is the presence of the third permanent molar (Teo et al. 2016; Ay et al. 2006; Murphy et al. 2022). Furthermore, a recent Norwegian study argued that long-term treatment, including cost and effort for the individual, must be considered when treatment decisions are made for FPMs with poor prognosis (Brusevold et al. 2021).

When choosing to keep and restore FPMs with MIH, resin composite and glass-ionomers cement (GIC) were the preferred materials. This is in concordance with other studies (Skaare et al. 2021; Crombie et al. 2008). In the present study in cases of moderate severity of MIH, the general dentists, as well as the pediatric dentists, chose to restore the FPMs with resin composite. However, in cases with severe MIH, the general dentists still preferred resin composite, while the pediatric dentists were more likely to choose GIC. The findings are comparable to a Spanish study, which also showed that pediatric dentists were more prone to choose GIC as a restorative material for FPMs with post-eruptive breakdown due to MIH (Serna-Muñoz et al. 2020). The difference in preferred materials may be due to the literature being varied, and that a patient's cooperation must be considered (Somani et al. 2022). An intact enamel surface is essential for bonding resin composite in order to avoid a high failure rate. Therefore, GIC may be a better alternative (Sönmez and Saat 2017).

Stainless-steel or porcelain crowns, inlay, or onlay as a treatment option for FPMs with MIH was an uncommon choice. In a Norwegian study, few respondents chose stainless-steel crowns (Uhlen et al. 2019). When comparing stainless-steel crowns with resin composite restoration, de Farias et al. (2022) found that the survival rate of the stainless-steel crowns was almost twice as high after 24 months. The respondents in this study chose extraction over stainless-steel crowns. Ceramic restoration has shown a high survival rate, compared to resin composite restoration (Linner et al. 2020). However, a Norwegian study showed that few dentists chose this type of treatment, which may be due to the inexperience of the respondents when handling prosthetics in children (Skaare et al. 2021). There is no basis on how many stainless-steel crowns are performed on children and adolescents in Sweden, but most dentists lack a tradition of performing stainless-steel crowns on children. When it comes to Ceramic restoration, the technology has developed and improved significantly in recent years. Unfortunately, there is a lack of randomized studies with long-term follow-ups.

When treating FPMs with mild MIH, the general dentists, as well as the pediatric dentists, chose fluoride varnish, while the orthodontists chose expectancy. This result is not unexpected as orthodontists are not involved in prophylactic dental care of patients with MIH. However, it has been shown that fluoride varnish is influential in diminishing hypersensibility and promotes a certain amount of remineralization (Kumar et al. 2022; Biondi et al. 2017; Fütterer et al. 2020).

In the present study, each FPM was graded as: intact tooth, mild MIH, moderate MIH, and severe MIH, according to disintegration of the enamel and number of affected surfaces. The EAPD’s criteria (Jälevik 2010; Lygidakis et al. 2022) was expanded with moderate MIH to facilitate data analysis.

The response rate of 36% entails difficulties in generalizing the results and increases the risk of bias. Nevertheless, compared to other studies with similar methods and fields of research, the response rate is equivalent (Serna-Muñoz et al. 2020; Gambetta-Tessini et al. 2016; Gamboa et al. 2018). The gender distribution of the responding general dentists corresponds to the gender distribution of dentists employed by the Public Dental Service in Region of Västra Götaland. A large proportion of Swedish orthodontists and pediatric dentists are members in each interest society. According to the Swedish National Board of Health and Welfare statistical database from the year 2020, 275 orthodontists and 123 pediatric dentists were employed (Socialstyrelsen 2022b). The higher number of members of the two associations indicates that there are members who are not employed, i.e., retired, and may be considered as a limitation of the sample selection. The response rate among the general dentists, orthodontists, and pediatric dentists is comparative and evenly distributed. A strength is that the respondents were spread over a large geographical area and treated children from different socio-economic and ethnic backgrounds.

Symptoms from affected teeth are crucial in determining the treatment choice. Symptom relief for the patient takes precedence, and both intra oral and extra oral statuses must be considered in treatment planning. In the clinical setting, the patient's temperament, level of maturity, and cooperation may influence the dentist. In this study, severity and technical difficulties were assessed based on clinical photos and radiographs for treatment selection. Consequently, the respondents had to indirectly evaluate the severity of MIH, with no information on symptoms, compared to studies where respondents were informed on the patient’s symptoms (Alkadhimi et al. 2022; Wall and Leith 2020). However, the absence of anamnestic information may be a limitation, as anamnesis, together with the clinical picture, is fundamental for therapy planning in real-life situations. Furthermore, the patient’s/caregiver’s wishes and values may influence treatment choices.

Managing FPMs with MIH is challenging, and the treatment plans should be adapted to the patient's needs. The uniqueness of this study is the focus on clinical assessment and therapy choice of FPMs with MIH. There is a need for randomized control trials with long-time follow-ups when treating FPMs with MIH. This is in agreement with da Costa Rosa et al. (2022).

## Conclusion

Considering any limitations of the present questionnaire study in the evaluated group of Swedish dentists it has been shown that:

- Extraction of first permanent molars with moderate and severe MIH appears to be the treatment of choice among general dentists and specialists, with the preferred time for extraction before the eruption of the second permanent molar. Choosing extraction of first permanent molars with severe MIH was significantly more common in the maxilla compared to the mandible.
- When restorative treatment was chosen for the moderately affected first permanent molars, composite resin restorations were preferred, while fluoride varnish application was chosen in cases of mild MIH.
- The general dentists find support in consulting mainly pediatric dentists before choosing treatment of first permanent molars with MIH.

### Supplementary Information

Below is the link to the electronic supplementary material.

Supplementary file1 (PDF 20351 kb)

Supplementary file2 (PDF 19121 kb)

Supplementary file3 (PDF 20350 kb)

Supplementary file4 (DOCX 2565 kb)

Supplementary file5 (DOCX 2565 kb)

Supplementary file6 (DOCX 2565 kb)

## Data Availability

Data and material the data used for this research can be made available upon request to the authors.
