# Supplementary material for: Treatment choice for first permanent molars affected with molar-incisor hypomineralization, in patients 7–8 years of age: a questionnaire study among Swedish general dentists, orthodontists, and pediatric dentists
Source: Eur Arch Paediatr Dent. 2024 Feb 5;25(1):93–103. doi: 10.1007/s40368-023-00860-9 (PMC10942915; doi:10.1007/s40368-023-00860-9)
Supplement: Supplementary file 3 — Supplementary file3 (PDF 20350 kb) [file 40368_2023_860_MOESM3_ESM.pdf]

Frågor om behandling av tänder med mineraliseringsstörning och sönderfall

Medlemmar i svenska pedodontiföreningen är utvalda för att besvara denna enkät.

Nedan följer några fall med tänder som drabbats av sönderfall till följd av mineraliseringsstörning. I varje fall ombeds du svara på vilken behandling du väljer. Fallen är från barn 7-8 år. Som underlag till varje fall finns kliniska foto, panoramaröntgen, profilröntgen samt foto på studiemodeller. Ingen ytterligare information om patienten delges, i syfte att hitta verktyg för behandlingsstrategier baserat på omfattning av tandsönderfall.
Det tar ca 15 minuter att besvara frågorna.

Dina svar är helt anonyma.
Stort tack för dina svar!

Nina Sabel, Agneta Robertson, Birgitta Jälevik, Emina Čirgić och Adnan Hajdarević

*Obligatorisk

GÖTEBORGS UNIVERSITET

VÄSTRA GÖTALANDSREGIONEN FOLKTANDVÅRDEN

Frågor om dig som besvarar frågorna:

1. 1. Vilket år fick du din tandläkarexamen? *

____

2. 2. Vilket år fick du din specialistexamen? *

____

3. 3. Vilket kön tillhör du? *

Markera endast en oval.

- Kvinna
- Man
- Vill ej ange

4. 4. Hur många timmar per vecka arbetar du kliniskt med barn och ungdomstandvård? *

Markera endast en oval.

- < 10 timmar
- 10-19 timmar
- 20-30 timmar
- > 30 timmar
- Inte kliniskt verksam

FALL 1

FALL 1

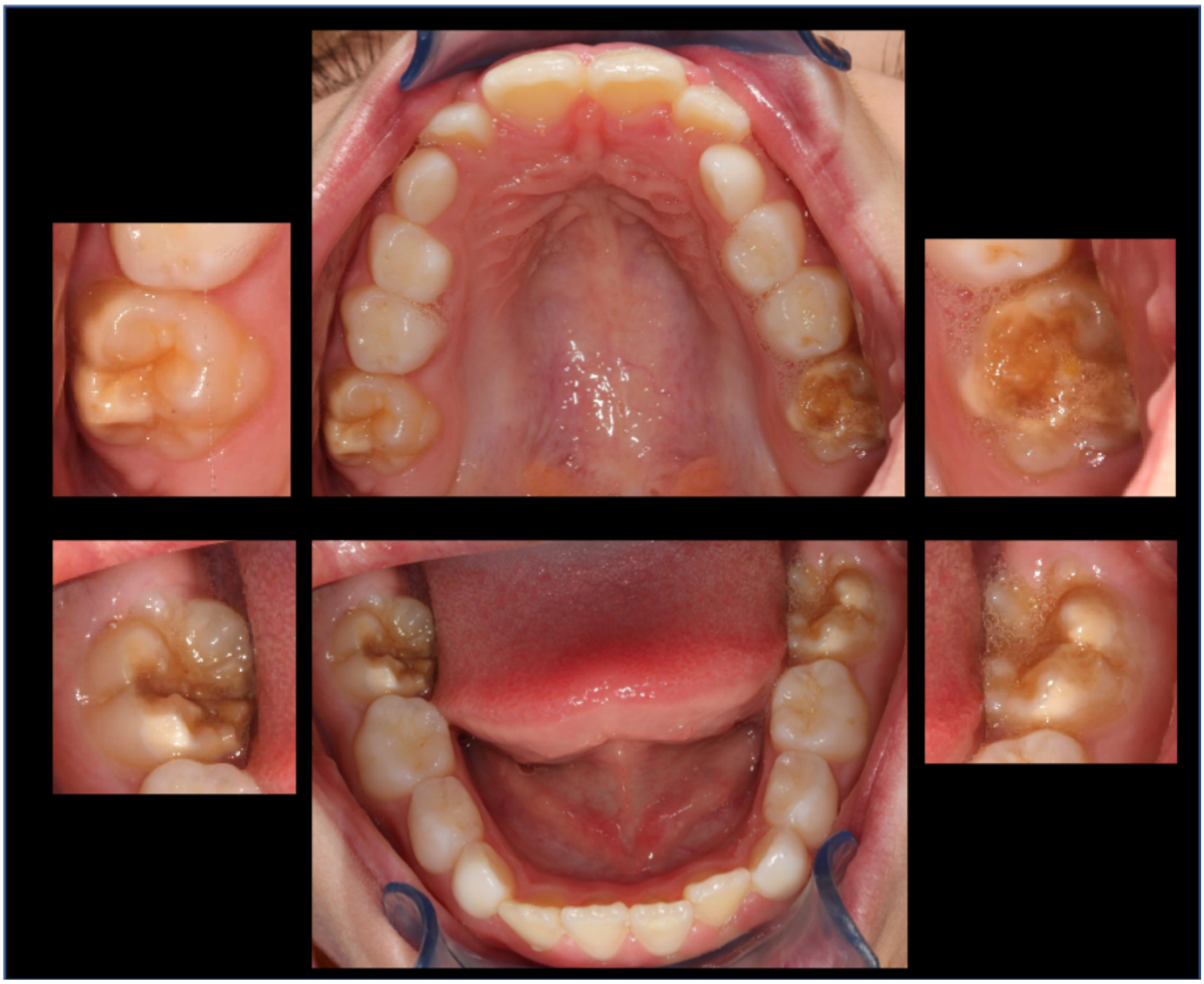

FALL 1

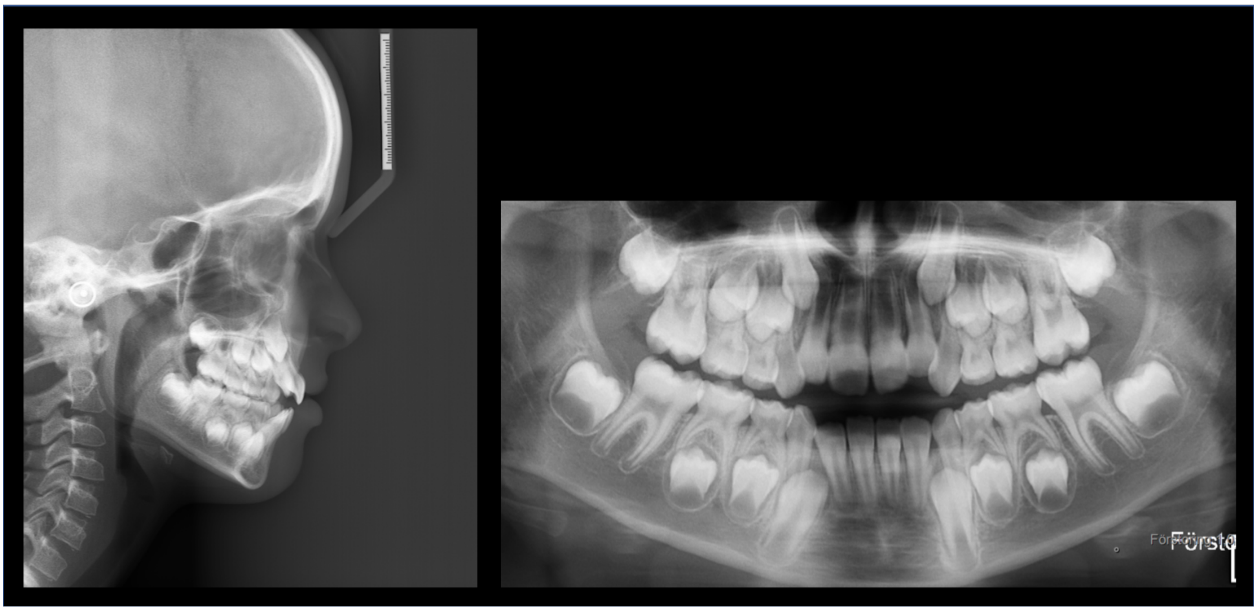

FALL 1

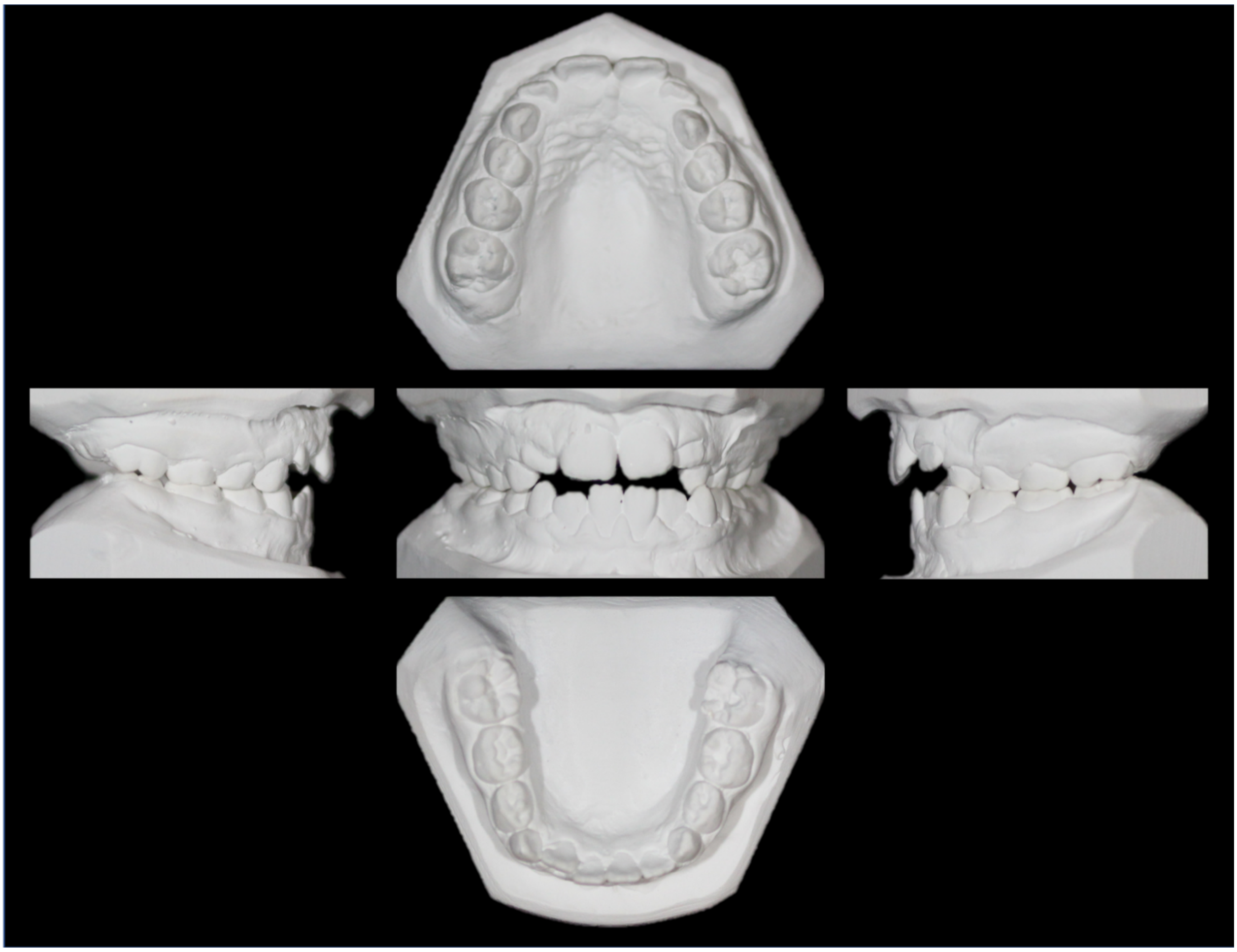

5. 1.1 Vilken behandling väljer du för tand 16? *

Markera endast en oval.

- Lagning med komposit
- Lagning med glasjonomer
- Stålkrona
- Kron- eller inläggsterapi
- Extraktion
- Utökad fluorbehandling
- Ekpektans

6. 1.2 Vilken behandling väljer du för tand 26? *

Markera endast en oval.

- Lagning med komposit
- Lagning med glasjonomer
- Stålkrona
- Kron- eller inläggsterapi
- Extraktion
- Utökad fluorbehandling
- Expektans

7. 1.3 Vilken behandling väljer du för tand 36? *

Markera endast en oval.

- Lagning med komposit
- Lagning med glasjonomer
- Stålkrona
- Kron- eller inläggsterapi
- Extraktion
- Utökad fluorbehandling
- Expektans

8. 1.4 Vilken behandling väljer du för tand 46? *

Markera endast en oval.

- Lagning med komposit
- Lagning med glasjonomer
- Stålkrona
- Kron- eller inläggsterapi
- Extraktion
- Utökad fluorbehandling
- Expektans

FALL 2

FALL 2

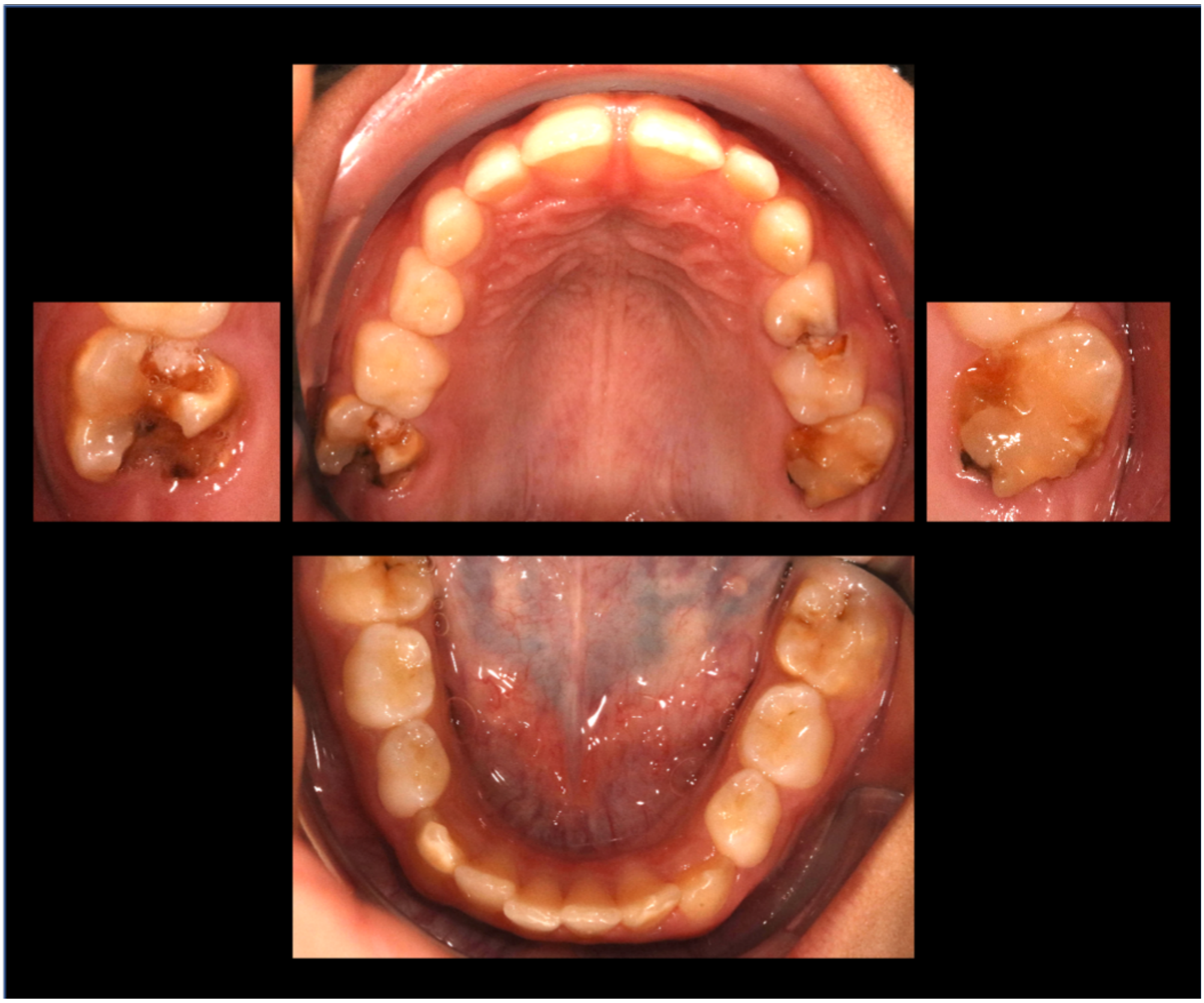

FALL 2

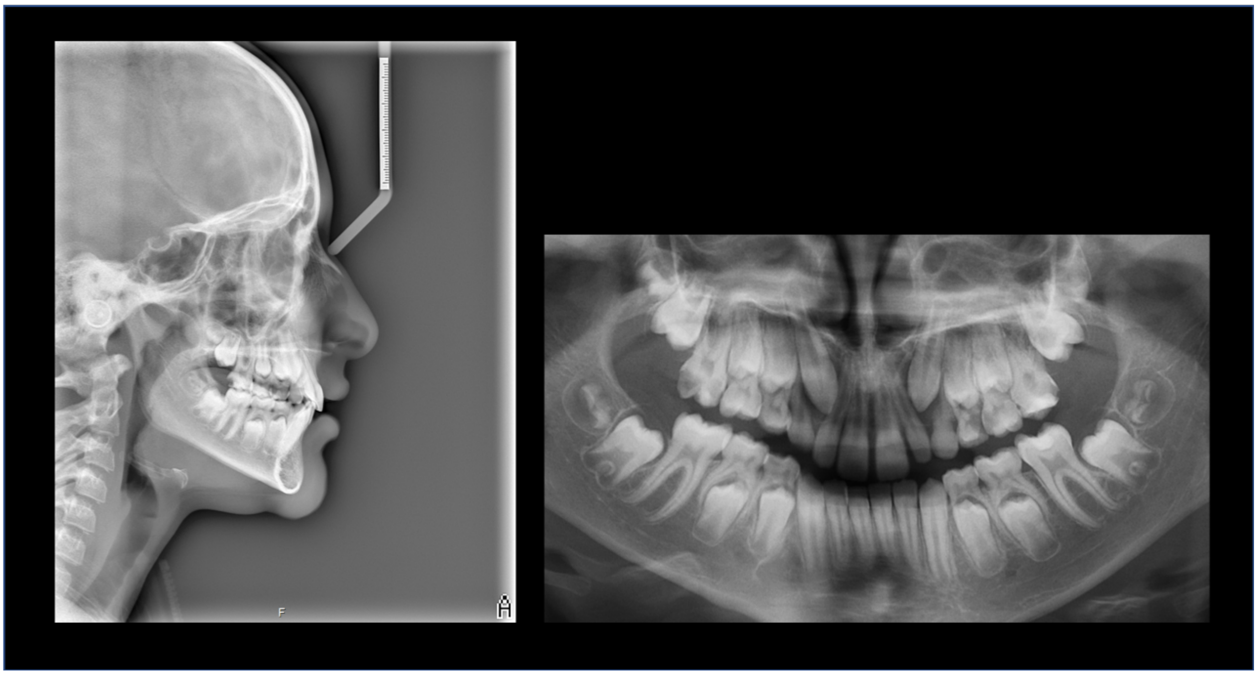

FALL 2

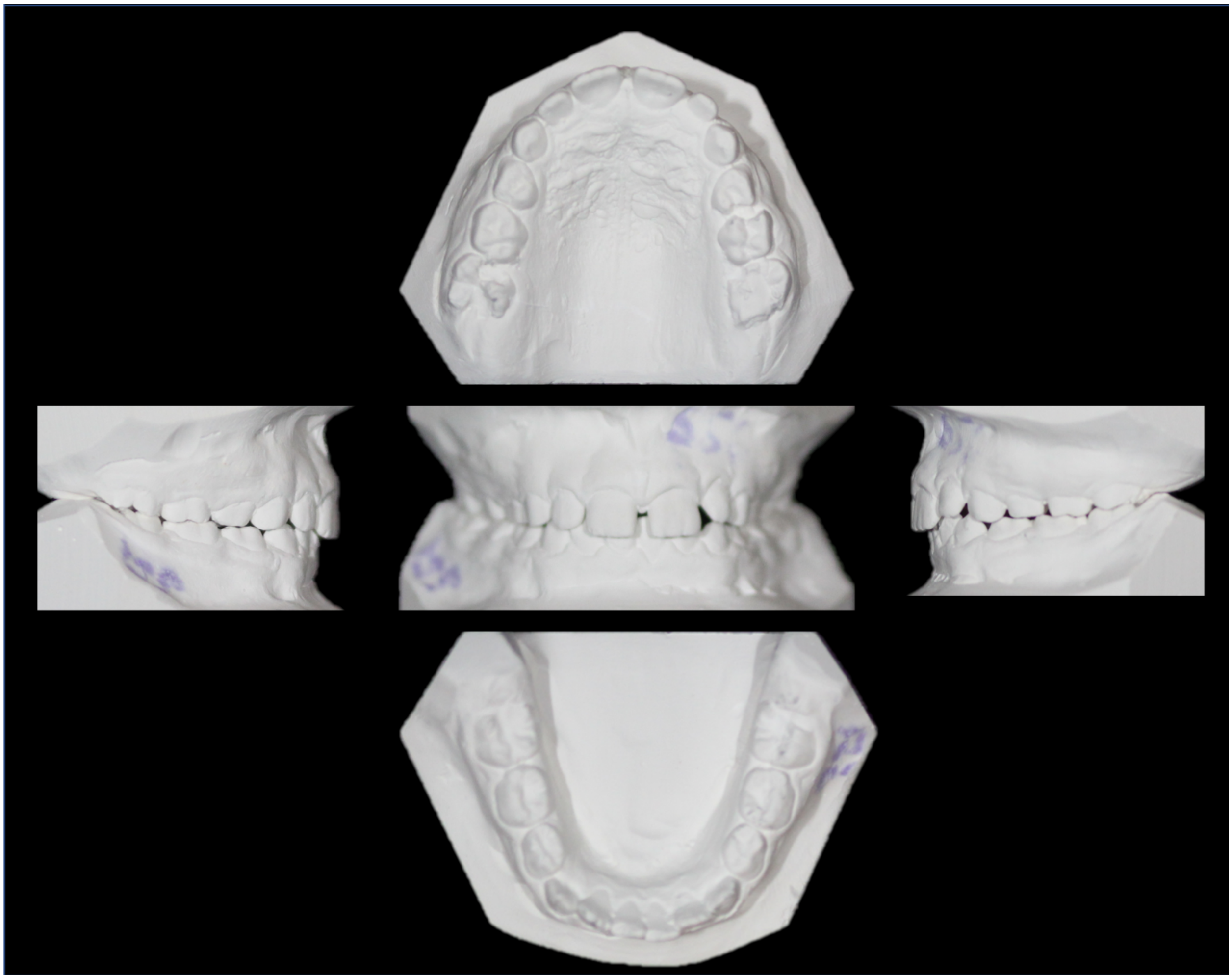

9. 2.1 Vilken behandling väljer du för tand 16? *

Markera endast en oval.

- Lagning med komposit
- Lagning med glasjonomer
- Stålkrona
- Kron- eller inläggsterapi
- Extraktion
- Utökad fluorbehandling
- Ekpekatns

10. 2.2 Vilken behandling väljer du för tand 26? *

Markera endast en oval.

- Lagning med komposit
- Lagning med glasjonomer
- Stålkrona
- Kron- eller inläggsterapi
- Extraktion
- Utökad fluorbehandling
- Expektans

11. 2.3 Vilken behandling väljer du för tand 36? *

Markera endast en oval.

- Lagning med komposit
- Lagning med glasjonomer
- Stålkrona
- Kron- eller inläggsterapi
- Extraktion
- Utökad fluorbehandling
- Expektans

12. 2.4 Vilken behandling väljer du för tand 46? *

Markera endast en oval.

- Lagning med komposit
- Lagning med glasjonomer
- Stålkrona
- Kron- eller inläggsterapi
- Extraktion
- Utökad fluorbehandling
- Expektans

FALL 3

FALL 3

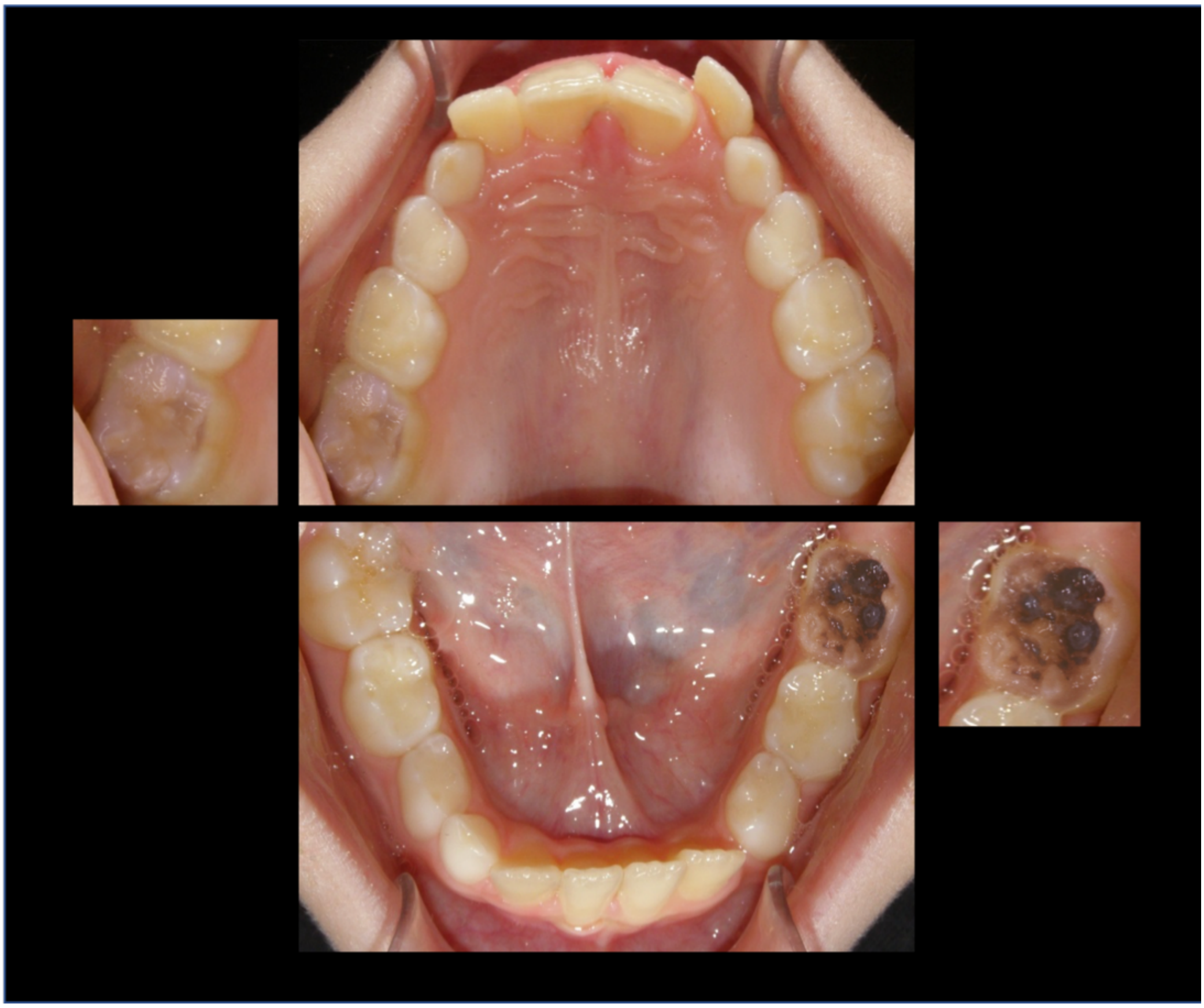

FALL 3

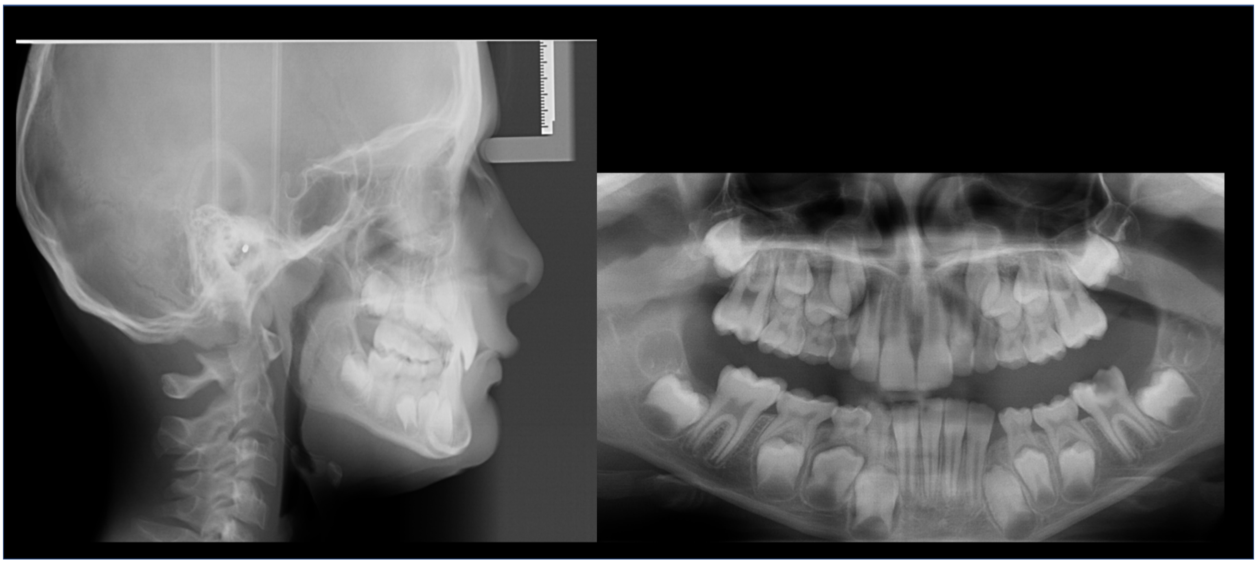

FALL 3

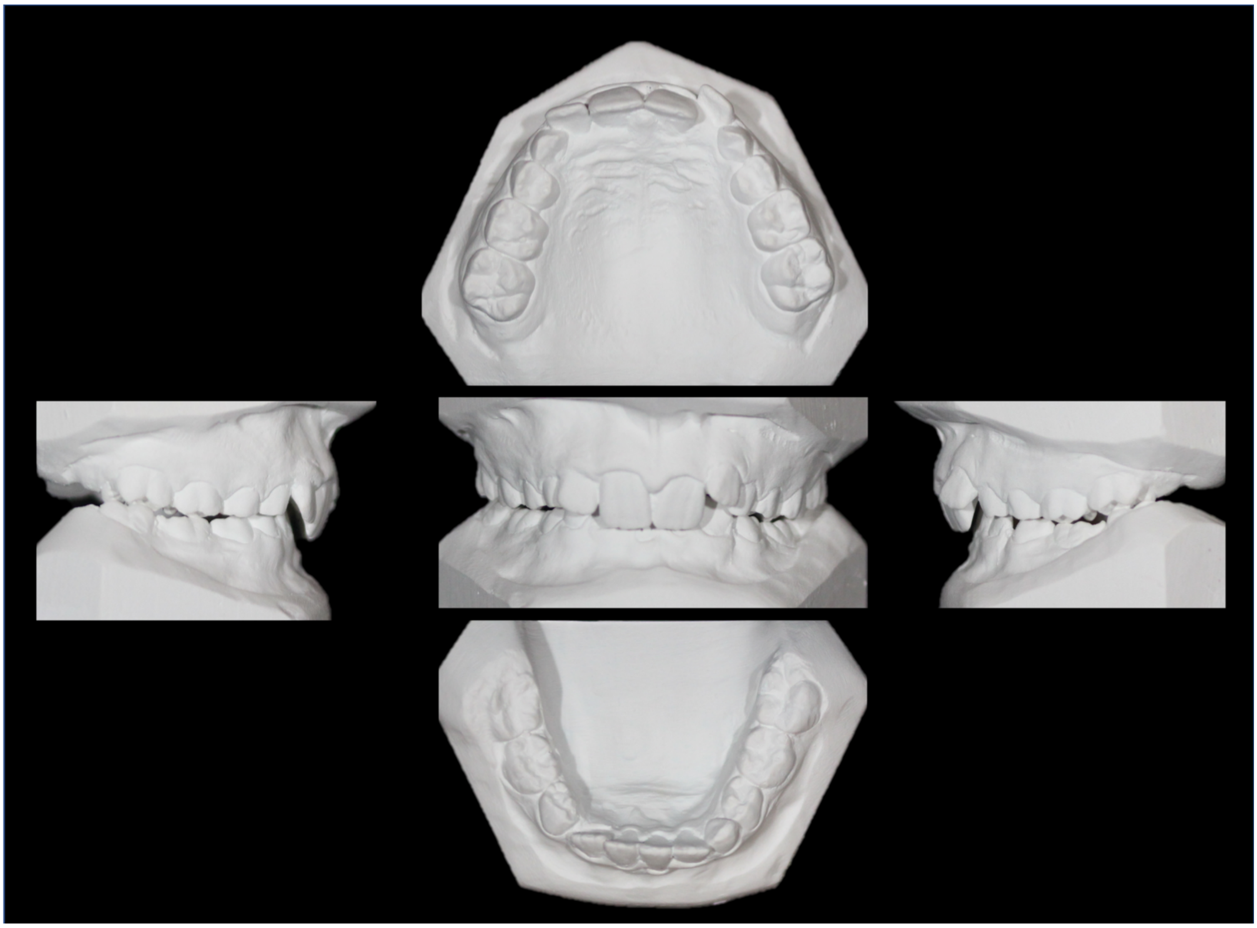

13. 3.1 Vilken behandling väljer du för tand 16? *

Markera endast en oval.

- Lagning med komposit
- Lagning med glasjonomer
- Stålkrona
- Kron- eller inläggsterapi
- Extraktion
- Utökad fluorbehandling
- Expektans

14. 3.2 Vilken behandling väljer du för tand 26? *

Markera endast en oval.

- Lagning med komposit
- Lagning med glasjonomer
- Stålkrona
- Kron- eller inläggsterapi
- Extraktion
- Utökad fluorbehandling
- Expektans

15. 3.3 Vilken behandling väljer du för tand 36? *

Markera endast en oval.

- Lagning med komposit
- Lagning med glasjonomer
- Stålkrona
- Kron- eller inläggsterapi
- Extraktion
- Utökad fluorbehandling
- Expektans

16. 3.4 Vilken behandling väljer du för tand 46? *

Markera endast en oval.

- Lagning med komposit
- Lagning med glasjonomer
- Stålkrona
- Kron- eller inläggsterapi
- Extraktion
- Utökad fluorbehandling
- Expektans

FALL 4

FALL 4

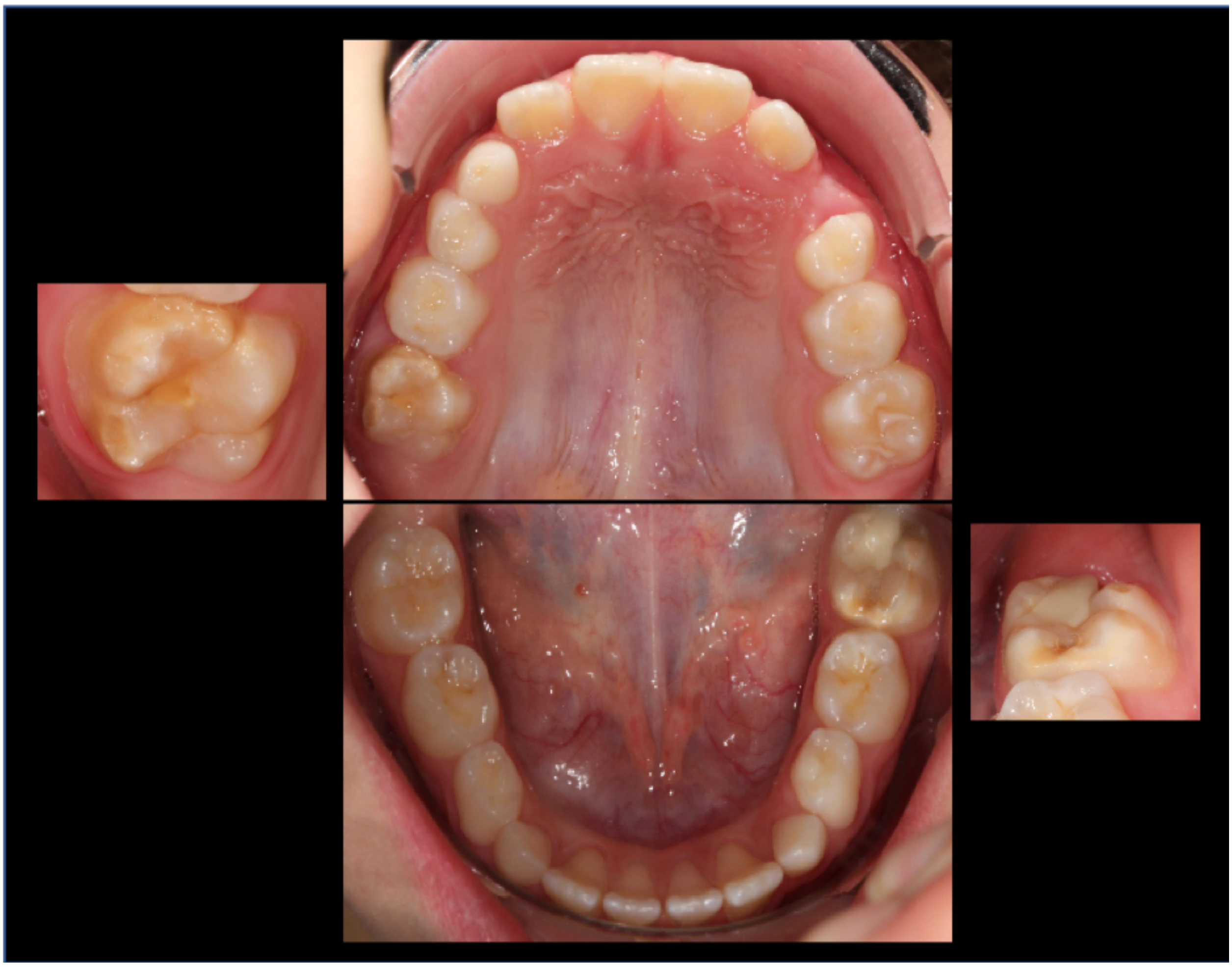

FALL 4

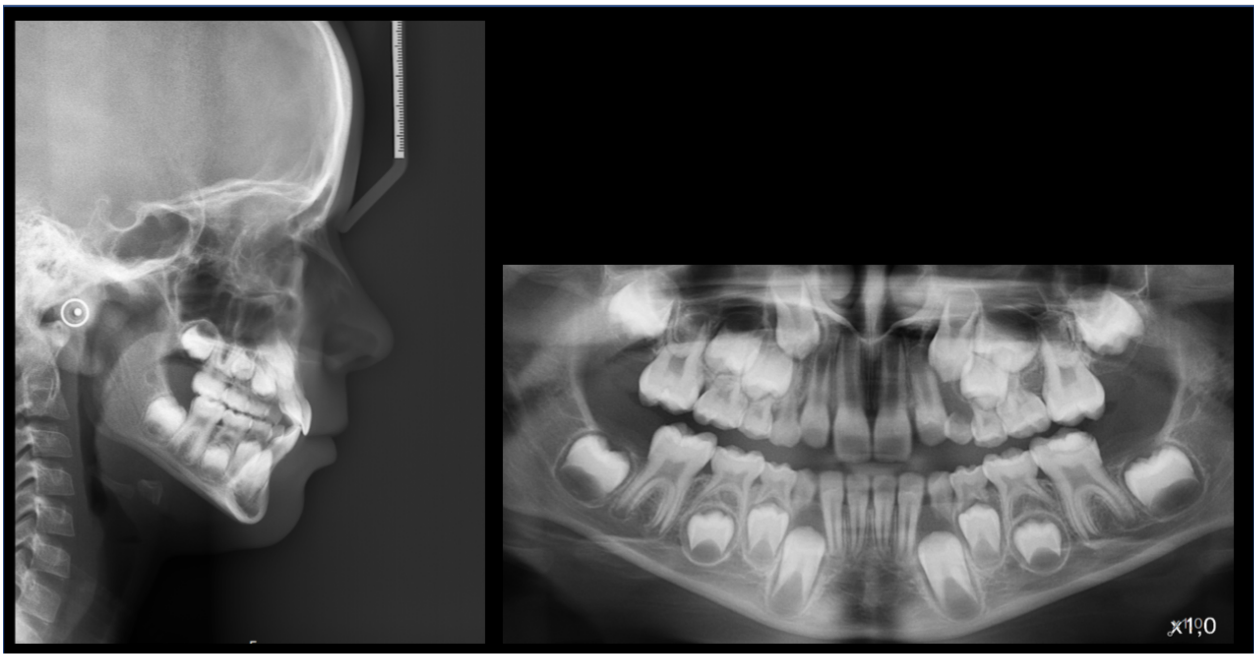

FALL 4

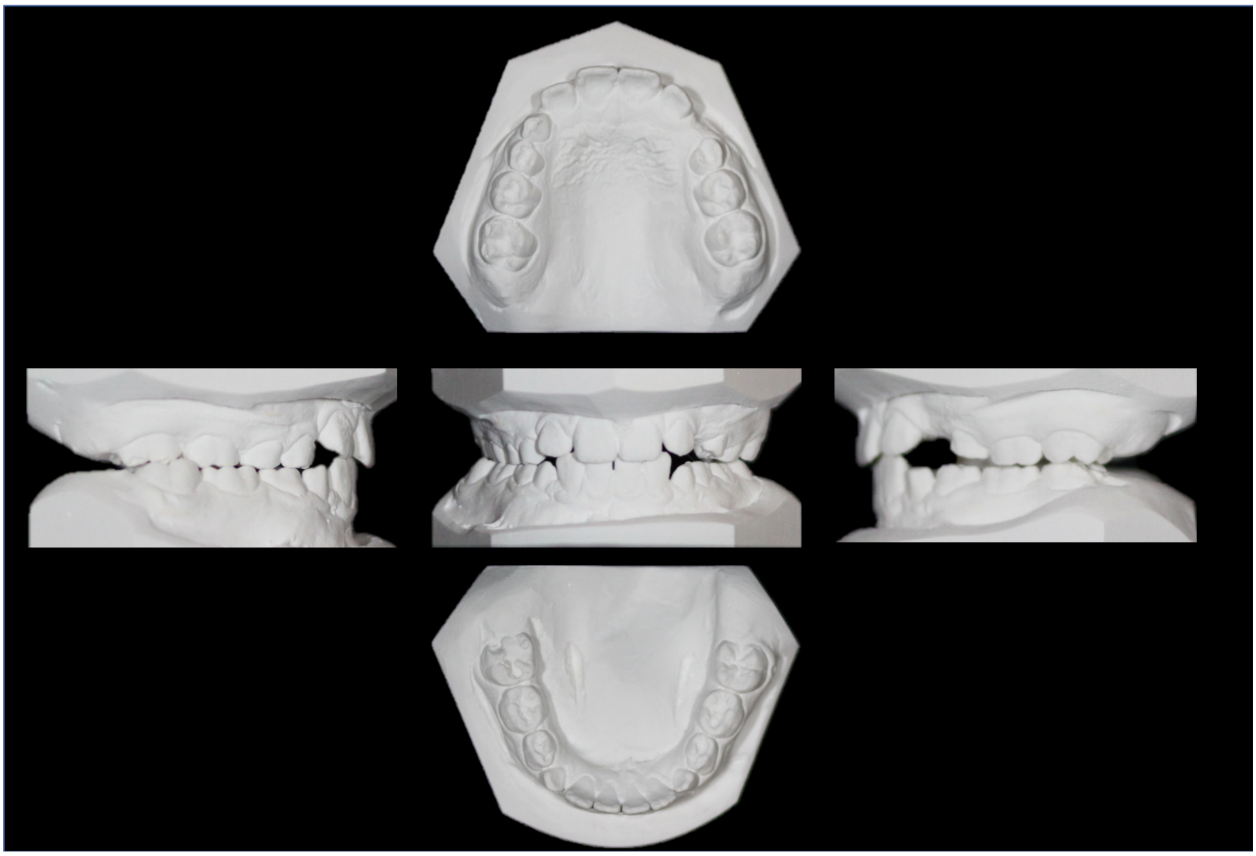

17. 4.1 Vilken behandling väljer du för tand 16? *

Markera endast en oval.

- Lagning med komposit
- Lagning med glasjonomer
- Stålkrona
- Kron- eller inläggsterapi
- Extraktion
- Utökad fluorbehandling
- Expektans

18. 4.2 Vilken behandling väljer du för tand 26? *

Markera endast en oval.

- Lagning med komposit
- Lagning med glasjonomer
- Stålkrona
- Kron- eller inläggsterapi
- Extraktion
- Utökad fluorbehandling
- Expektans

19. 4.3 Vilken behandling väljer du för tand 36? *

Markera endast en oval.

- Lagning med komposit
- Lagning med glasjonomer
- Stålkrona
- Kron- eller inläggsterapi
- Extraktion
- Utökad fluorbehandling
- Expektans

20. 4.4 Vilken behandling väljer du för tand 46? *

Markera endast en oval.

- Lagning med komposit
- Lagning med glasjonomer
- Stålkrona
- Kron- eller inläggsterapi
- Extraktion
- Utökad fluorbehandling
- Expektans

FALL 5

FALL 5

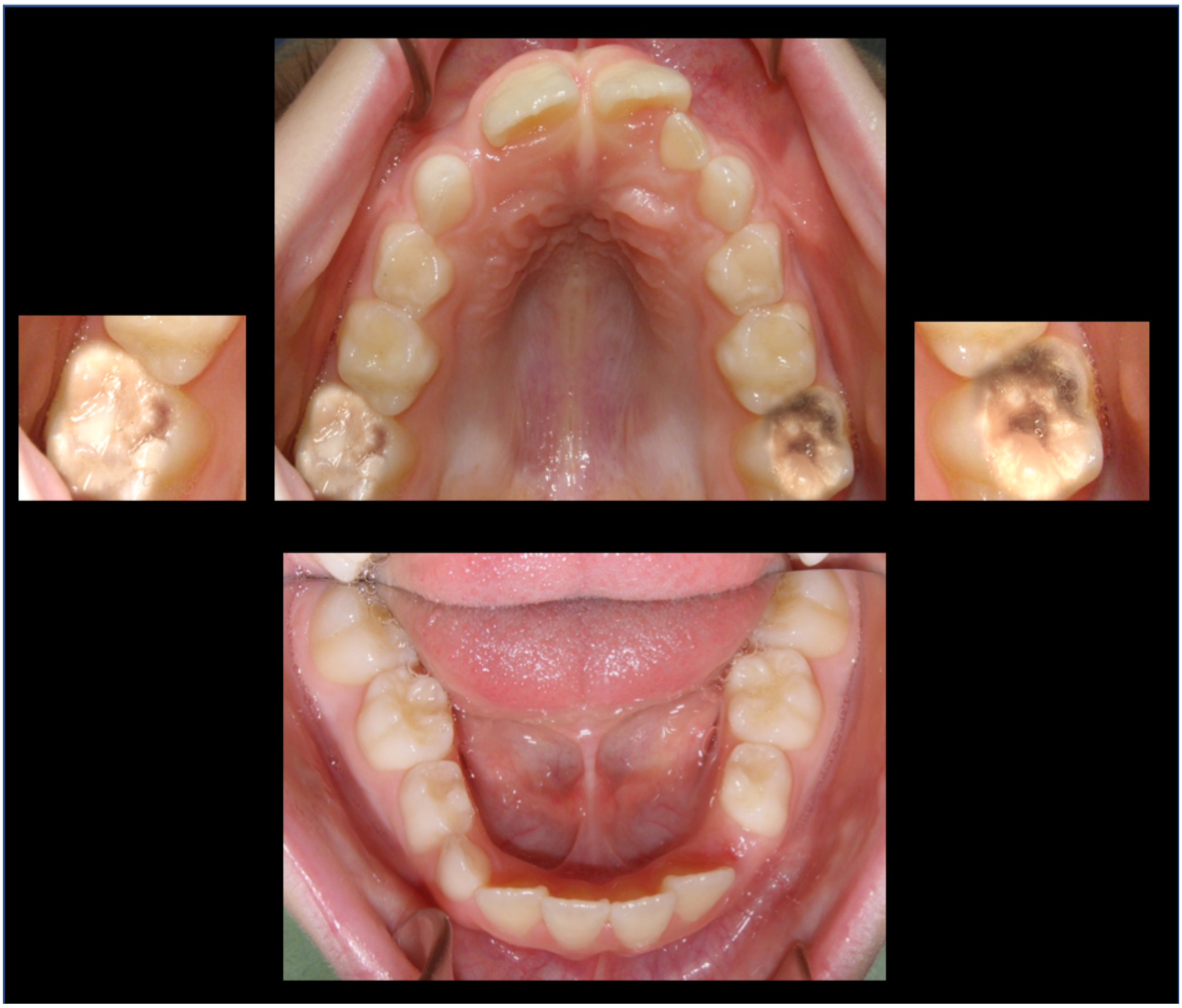

FALL 5

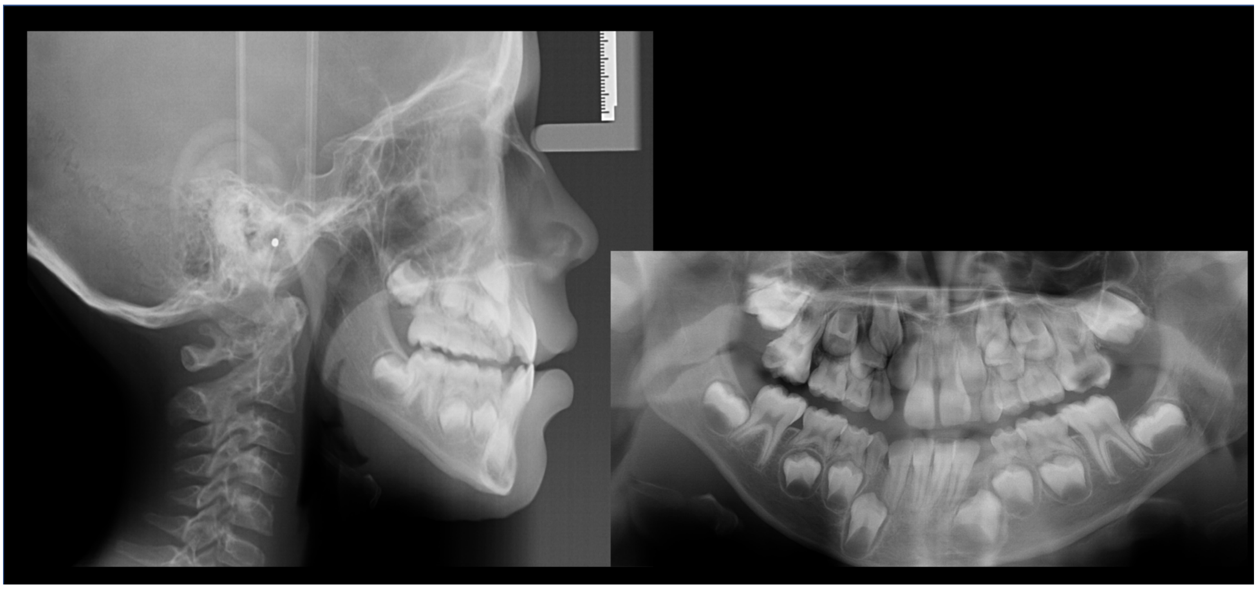

FALL 5

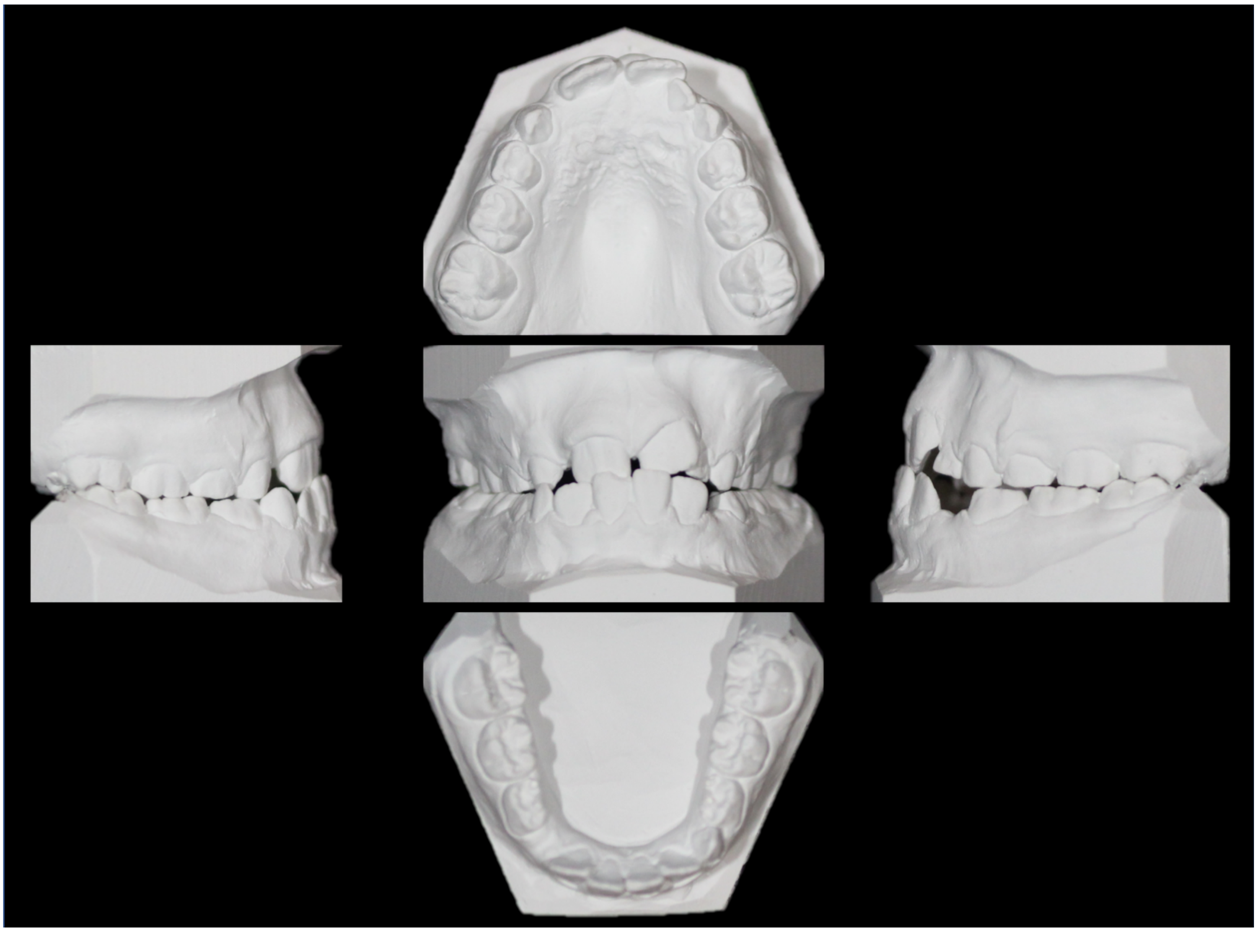

21. 5.1 Vilken behandling väljer du för tand 16? *

Markera endast en oval.

- Lagning med komposit
- Lagning med glasjonomer
- Stålkrona
- Kron- eller inläggsterapi
- Extraktion
- Utökad fluorbehandling
- Expektans

22. 5.2 Vilken behandling väljer du för tand 26? *

Markera endast en oval.

- Lagning med komposit
- Lagning med glasjonomer
- Stålkrona
- Kron- eller inläggsterapi
- Extraktion
- Utökad fluorbehandling
- Expektans

23. 5.3 Vilken behandling väljer du för tand 36? *

Markera endast en oval.

- Lagning med komposit
- Lagning med glasjonomer
- Stålkrona
- Kron- eller inläggsterapi
- Extraktion
- Utökad fluorbehandling
- Expektans

24. 5.4 Vilken behandling väljer du för tand 46? *

Markera endast en oval.

- Lagning med komposit
- Lagning med glasjonomer
- Stålkrona
- Kron- eller inläggsterapi
- Extraktion
- Utökad fluorbehandling
- Expektans

FALL 6

FALL 6

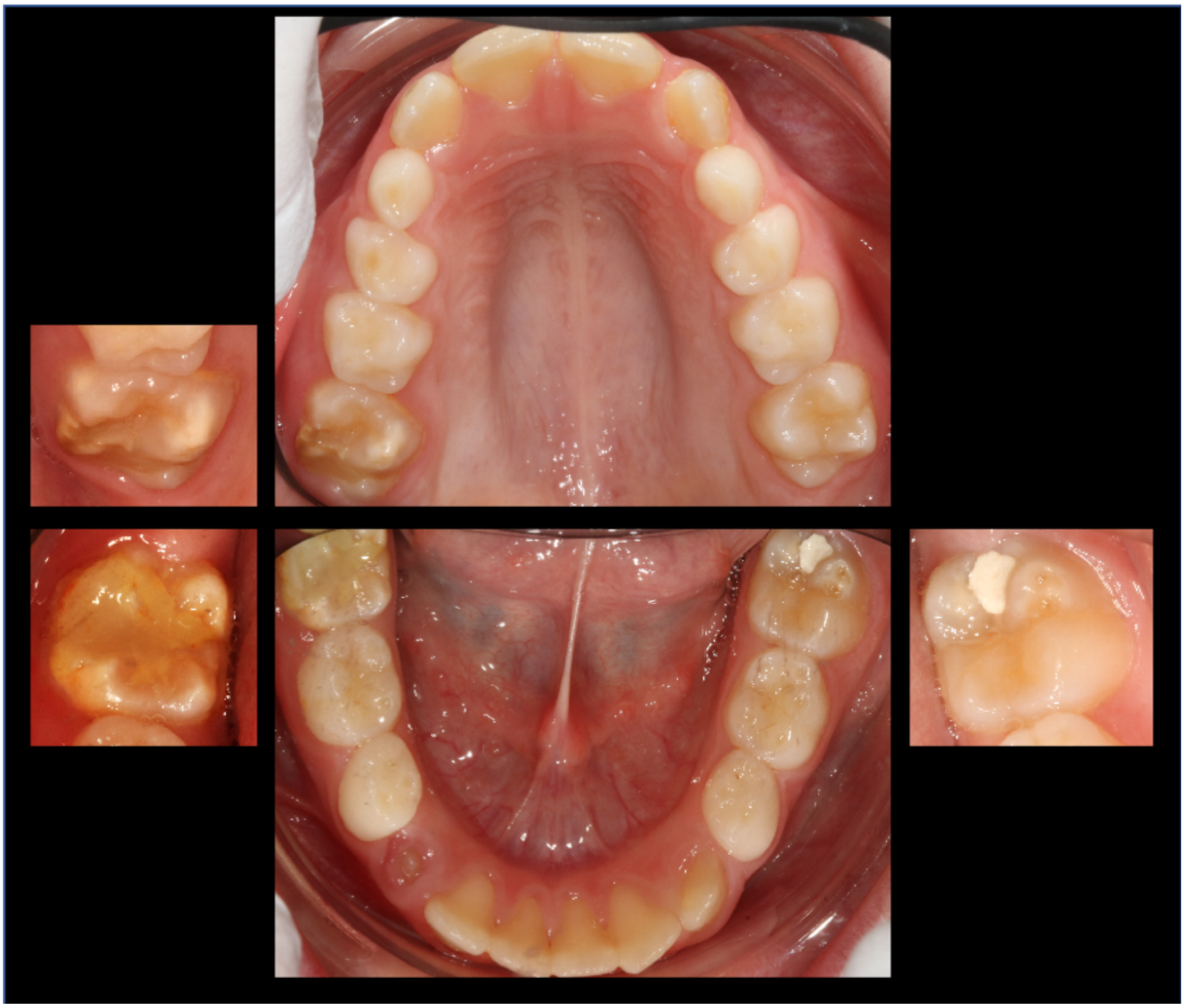

FALL 6

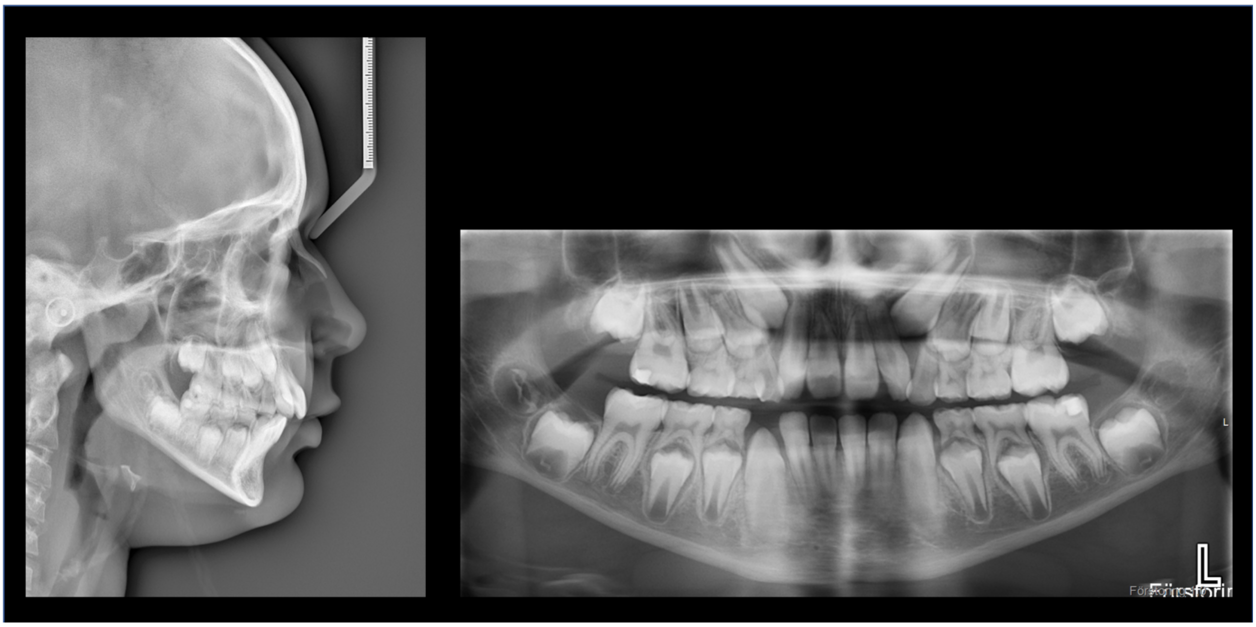

FALL 6

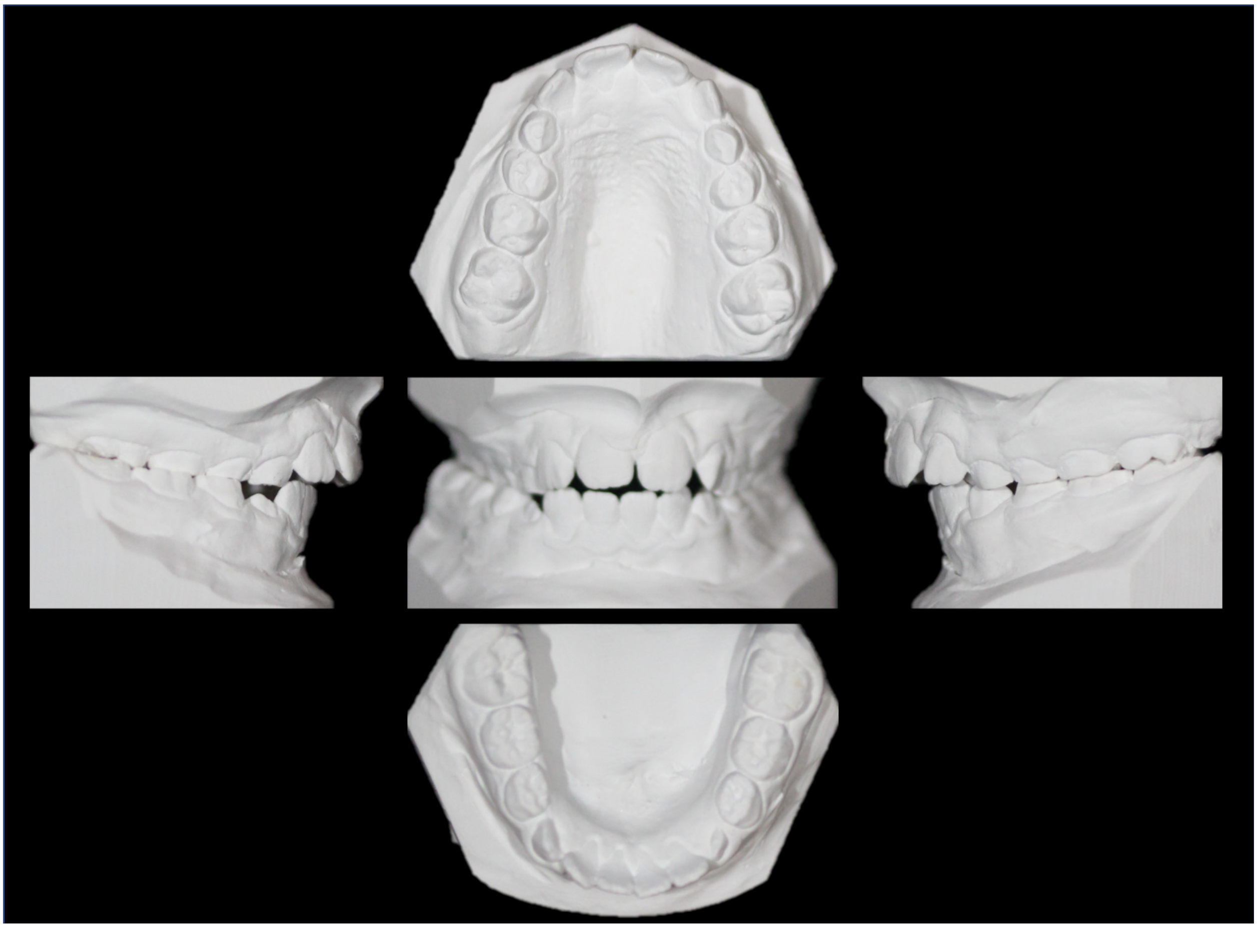

25. 6.1 Vilken behandling väljer du för tand 16? *

Markera endast en oval.

- Lagning med komposit
- Lagning med glasjonomer
- Stålkrona
- Kron- eller inläggsterapi
- Extraktion
- Utökad fluorbehandling
- Expektans

26. 6.2 Vilken behandling väljer du för tand 26? *

Markera endast en oval.

- Lagning med komposit
- Lagning med glasjonomer
- Stålkrona
- Kron- eller inläggsterapi
- Extraktion
- Utökad fluorbehandling
- Expektans

27. 6.3 Vilken behandling väljer du för tand 36? *

Markera endast en oval.

- Lagning med komposit
- Lagning med glasjonomer
- Stålkrona
- Kron- eller inläggsterapi
- Extraktion
- Utökad fluorbehandling
- Expektans

28. 6.4 Vilken behandling väljer du för tand 46? *

Markera endast en oval.

- Lagning med komposit
- Lagning med glasjonomer
- Stålkrona
- Kron- eller inläggsterapi
- Extraktion
- Utökad fluorbehandling
- Expektans

Generella frågor

29. 11.1 Om du väljer extraktion av 6:a med gravt sönderfall, när utför du den? *

Markera endast en oval.

- Direkt vid diagnos
- Vid ca 8-9 års ålder?
- När man ser 7:ans rotfurkation (rotutveckling) på röntgen
- När 7:an börjar eruptera
- När 7:an är i ocklusion
- Övrigt: ____________________

30. 11.2 Vad baserar du oftast dina beslut på, konserverande behandling eller extraktion, vid grav MIH? *

Markera endast en oval.

- Klinisk erfarenhet
- Forskning
- Lokala riktlinjer
- Rekommendation av ortodontist

TACK FÖR DINA SVAR! Komihåg att trycka på knappen "skicka".

GÖTEBORGS UNIVERSITET

VÄSTRA GÖTALANDSREGIONEN FOLKTANDVÅRDEN

Det här innehållet har varken skapats eller godkänts av Google.

Google Formulär
