# Supplementary material for: Treatment choice for first permanent molars affected with molar-incisor hypomineralization, in patients 7–8 years of age: a questionnaire study among Swedish general dentists, orthodontists, and pediatric dentists
Source: Eur Arch Paediatr Dent. 2024 Feb 5;25(1):93–103. doi: 10.1007/s40368-023-00860-9 (PMC10942915; doi:10.1007/s40368-023-00860-9)
Supplement: Supplementary file 4 — Supplementary file4 (DOCX 2565 kb) [file 40368_2023_860_MOESM4_ESM.docx]

# QUESTIONNAIRE REGARDING THE TREATMENT OF TEETH WITH MINERALIZATION DISORDERS WITH POST-ERUPTIVE BREAKDOWN

General dentists working at the Public Dental Service in Region Västra Götaland are selected to answer this survey; participation in the survey is anchored via the management of the Public Dental Service.

Below are some cases of teeth that have suffered post-eruptive breakdown due to mineralization disorder. In each case, you are asked to answer which treatment you choose. The cases are from children 7-8 years old. The basis for each case: Clinical photo, panoramic radiographs, profile radiographs, and photos of study models. No additional information regarding the patient is given, with the aim to find tools for treatment strategies based on the extent of tooth breakdown.

It takes about 20 minutes to answer the questions.

Your answers are completely anonymous.

Thank you for your answers!

Agneta Robertson, Birgitta Jälevik, Emina Čirgić, Nina Sabel and Adnan Hajdarević

*Compulsory


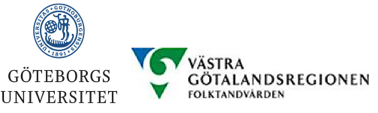


## QUESTIONS ABOUT YOU ANSWERING THIS QUESTIONNAIRE:

1. What year did you get your dental degree? *
2. What gender do you belong to? *
   - Female
   - Male
   - Do not wish to specify
3. How many hours per week do you clinically work with children and young people? *
   - <10
   - 10–19
   - 20–30
   - >30
   - Do not work clinically

CASE 1

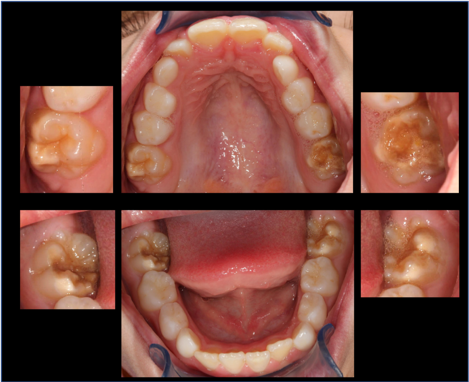


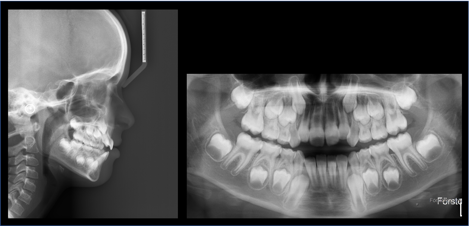


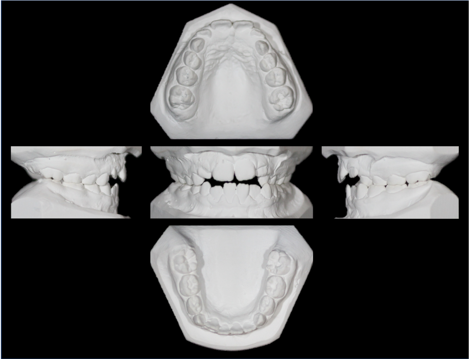


1.1 Which treatment do you choose for tooth 16? *
*Select only one*

- Restoration with composite resin
- Restoration with glass ionomer cement
- Stainless steel crown
- Porcelain crown, inlay or onlay
- Extraction
- Fluoride varnish
- Expectancy

1.2 Which treatment do you choose for tooth 26? *
*Select only one*

- Restoration with composite resin
- Restoration with glass ionomer cement
- Stainless steel crown
- Porcelain crown, inlay or onlay
- Extraction
- Fluoride varnish
- Expectancy

1.3 Which treatment do you choose for tooth 36? *
*Select only one*

- Restoration with composite resin
- Restoration with glass ionomer cement
- Stainless steel crown
- Porcelain crown, inlay or onlay
- Extraction
- Fluoride varnish
- Expectancy

1.4 Which treatment do you choose for tooth 46? *
*Select only one*

- Restoration with composite resin
- Restoration with glass ionomer cement
- Stainless steel crown
- Porcelain crown, inlay or onlay
- Extraction
- Fluoride varnish
- Expectancy

CASE 2

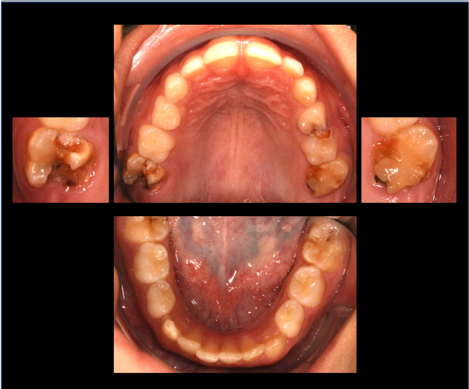


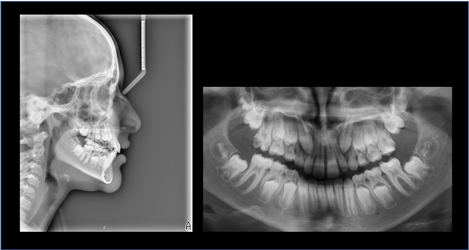


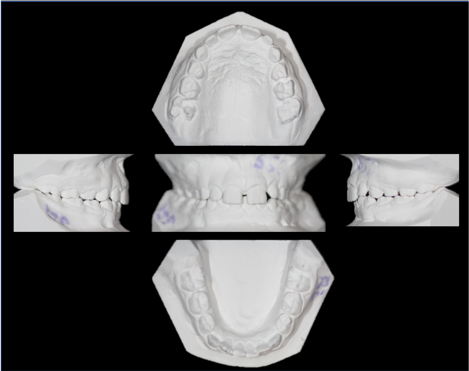


2.1 Which treatment do you choose for tooth 16? *
*Select only one*

- Restoration with composite resin
- Restoration with glass ionomer cement
- Stainless steel crown
- Porcelain crown, inlay or onlay
- Extraction
- Fluoride varnish
- Expectancy

2.2 Which treatment do you choose for tooth 26? *
*Select only one*

- Restoration with composite resin
- Restoration with glass ionomer cement
- Stainless steel crown
- Porcelain crown, inlay or onlay
- Extraction
- Fluoride varnish
- Expectancy

2.3 Which treatment do you choose for tooth 36? *
*Select only one*

- Restoration with composite resin
- Restoration with glass ionomer cement
- Stainless steel crown
- Porcelain crown, inlay or onlay
- Extraction
- Fluoride varnish
- Expectancy

2.4 Which treatment do you choose for tooth 46? *
*Select only one*

- Restoration with composite resin
- Restoration with glass ionomer cement
- Stainless steel crown
- Porcelain crown, inlay or onlay
- Extraction
- Fluoride varnish
- Expectancy

CASE 3

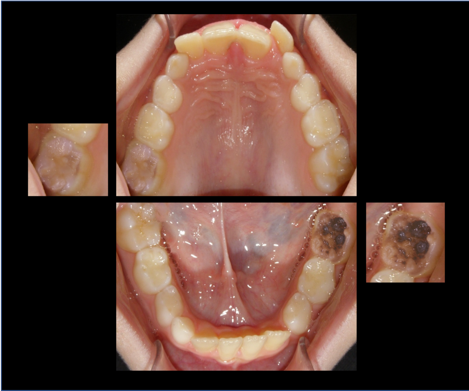


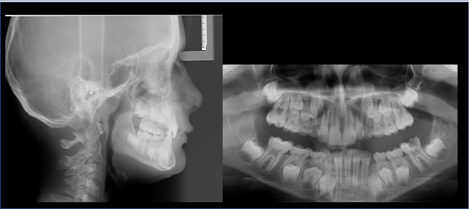


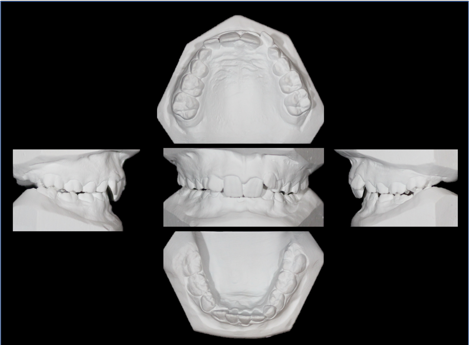


3.1 Which treatment do you choose for tooth 16? *
*Select only one*

- Restoration with composite resin
- Restoration with glass ionomer cement
- Stainless steel crown
- Porcelain crown, inlay or onlay
- Extraction
- Fluoride varnish
- Expectancy

3.2 Which treatment do you choose for tooth 26? *
*Select only one*

- Restoration with composite resin
- Restoration with glass ionomer cement
- Stainless steel crown
- Porcelain crown, inlay or onlay
- Extraction
- Fluoride varnish
- Expectancy

3.3 Which treatment do you choose for tooth 36? *
*Select only one*

- Restoration with composite resin
- Restoration with glass ionomer cement
- Stainless steel crown
- Porcelain crown, inlay or onlay
- Extraction
- Fluoride varnish
- Expectancy

3.4 Which treatment do you choose for tooth 46? *
*Select only one*

- Restoration with composite resin
- Restoration with glass ionomer cement
- Stainless steel crown
- Porcelain crown, inlay or onlay
- Extraction
- Fluoride varnish
- Expectancy

CASE 4

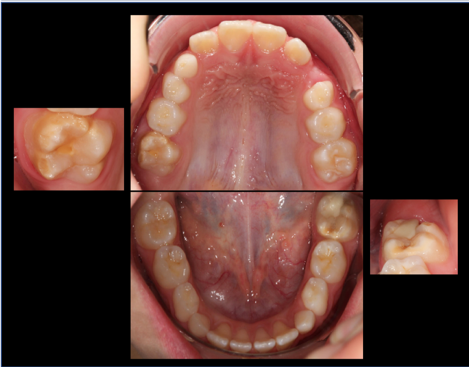


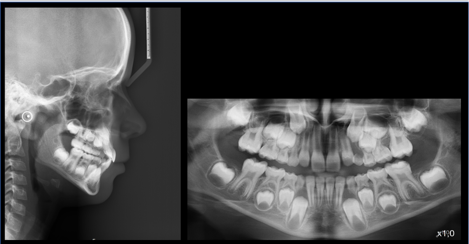


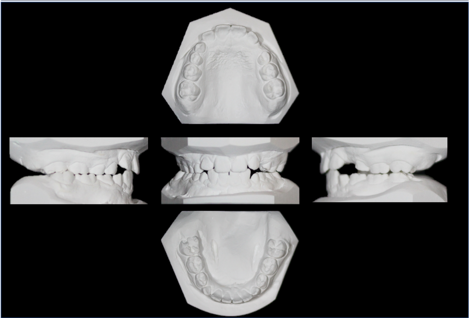


4.1 Which treatment do you choose for tooth 16? *
*Select only one*

- Restoration with composite resin
- Restoration with glass ionomer cement
- Stainless steel crown
- Porcelain crown, inlay or onlay
- Extraction
- Fluoride varnish
- Expectancy

4.2 Which treatment do you choose for tooth 26? *
*Select only one*

- Restoration with composite resin
- Restoration with glass ionomer cement
- Stainless steel crown
- Porcelain crown, inlay or onlay
- Extraction
- Fluoride varnish
- Expectancy

4.3 Which treatment do you choose for tooth 36? *
*Select only one*

- Restoration with composite resin
- Restoration with glass ionomer cement
- Stainless steel crown
- Porcelain crown, inlay or onlay
- Extraction
- Fluoride varnish
- Expectancy

4.4 Which treatment do you choose for tooth 46? *
*Select only one*

- Restoration with composite resin
- Restoration with glass ionomer cement
- Stainless steel crown
- Porcelain crown, inlay or onlay
- Extraction
- Fluoride varnish
- Expectancy

CASE 5

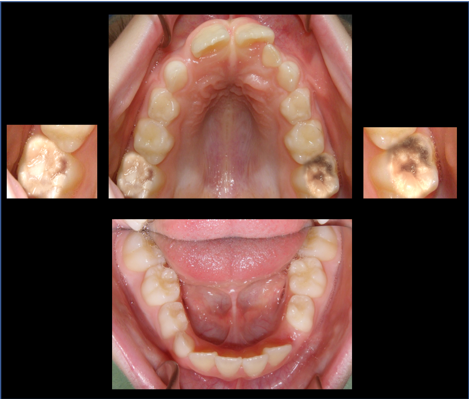


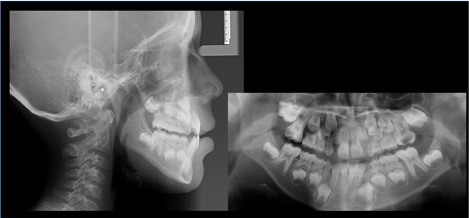


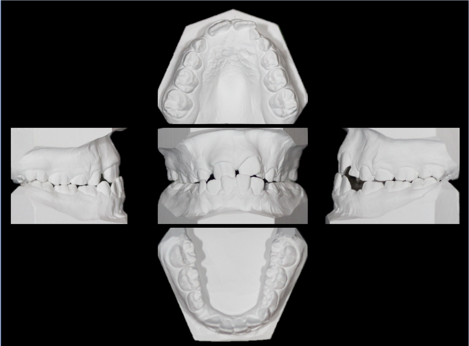


5.1 Which treatment do you choose for tooth 16? *
*Select only one*

- Restoration with composite resin
- Restoration with glass ionomer cement
- Stainless steel crown
- Porcelain crown, inlay or onlay
- Extraction
- Fluoride varnish
- Expectancy

5.2 Which treatment do you choose for tooth 26? *
*Select only one*

- Restoration with composite resin
- Restoration with glass ionomer cement
- Stainless steel crown
- Porcelain crown, inlay or onlay
- Extraction
- Fluoride varnish
- Expectancy

5.3 Which treatment do you choose for tooth 36? *
*Select only one*

- Restoration with composite resin
- Restoration with glass ionomer cement
- Stainless steel crown
- Porcelain crown, inlay or onlay
- Extraction
- Fluoride varnish
- Expectancy

5.4 Which treatment do you choose for tooth 46? *
*Select only one*

- Restoration with composite resin
- Restoration with glass ionomer cement
- Stainless steel crown
- Porcelain crown, inlay or onlay
- Extraction
- Fluoride varnish
- Expectancy

CASE 6

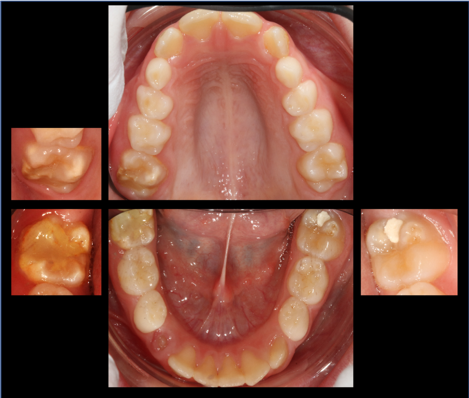


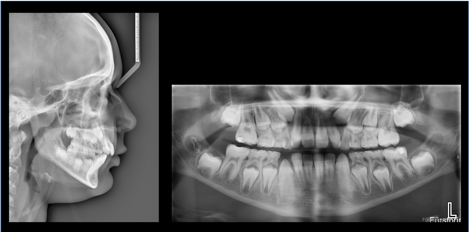


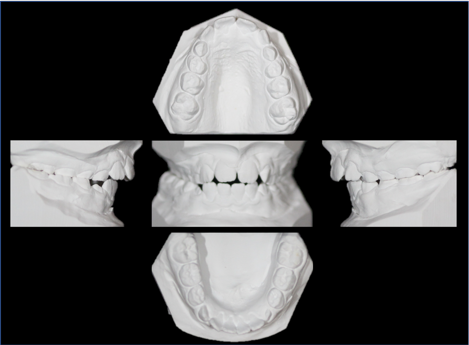


6.1 Which treatment do you choose for tooth 16? *
*Select only one*

- Restoration with composite resin
- Restoration with glass ionomer cement
- Stainless steel crown
- Porcelain crown, inlay or onlay
- Extraction
- Fluoride varnish
- Expectancy

6.2 Which treatment do you choose for tooth 26? *
*Select only one*

- Restoration with composite resin
- Restoration with glass ionomer cement
- Stainless steel crown
- Porcelain crown, inlay or onlay
- Extraction
- Fluoride varnish
- Expectancy

6.3 Which treatment do you choose for tooth 36? *
*Select only one*

- Restoration with composite resin
- Restoration with glass ionomer cement
- Stainless steel crown
- Porcelain crown, inlay or onlay
- Extraction
- Fluoride varnish
- Expectancy

6.4 Which treatment do you choose for tooth 46? *
*Select only one*

- Restoration with composite resin
- Restoration with glass ionomer cement
- Stainless steel crown
- Porcelain crown, inlay or onlay
- Extraction
- Fluoride varnish
- Expectancy

## GENERAL QUESTIONS

1. If you choose extraction of FPM with severe MIH and post-eruptive breakdown, when do you perform it? *
   *Select only one*

- Immediately upon diagnosis
- Between the age of 8-9 years old
- Root furcation development of second permanent molar on X-rays
- When the second permanent molar is partly erupted
- When the second permanent molar is fully erupted
- Other:___________

1. What do you usually base your treatment decision on while choosing conservative or extraction therapy of FPM with severe MIH? *
   *Select only one*

- Clinical experience
- Research
- Local guidelines
- Recommendation from the orthodontists
- Recommendation from the pediatric dentist
